# The Allelic Landscape of Human Blood Cell Trait Variation and Links to Common Complex Disease

**DOI:** 10.1016/j.cell.2016.10.042

**Published:** 2016-11-17

**Authors:** William J. Astle, Heather Elding, Tao Jiang, Dave Allen, Dace Ruklisa, Alice L. Mann, Daniel Mead, Heleen Bouman, Fernando Riveros-Mckay, Myrto A. Kostadima, John J. Lambourne, Suthesh Sivapalaratnam, Kate Downes, Kousik Kundu, Lorenzo Bomba, Kim Berentsen, John R. Bradley, Louise C. Daugherty, Olivier Delaneau, Kathleen Freson, Stephen F. Garner, Luigi Grassi, Jose Guerrero, Matthias Haimel, Eva M. Janssen-Megens, Anita Kaan, Mihir Kamat, Bowon Kim, Amit Mandoli, Jonathan Marchini, Joost H.A. Martens, Stuart Meacham, Karyn Megy, Jared O’Connell, Romina Petersen, Nilofar Sharifi, Simon M. Sheard, James R. Staley, Salih Tuna, Martijn van der Ent, Klaudia Walter, Shuang-Yin Wang, Eleanor Wheeler, Steven P. Wilder, Valentina Iotchkova, Carmel Moore, Jennifer Sambrook, Hendrik G. Stunnenberg, Emanuele Di Angelantonio, Stephen Kaptoge, Taco W. Kuijpers, Enrique Carrillo-de-Santa-Pau, David Juan, Daniel Rico, Alfonso Valencia, Lu Chen, Bing Ge, Louella Vasquez, Tony Kwan, Diego Garrido-Martín, Stephen Watt, Ying Yang, Roderic Guigo, Stephan Beck, Dirk S. Paul, Tomi Pastinen, David Bujold, Guillaume Bourque, Mattia Frontini, John Danesh, David J. Roberts, Willem H. Ouwehand, Adam S. Butterworth, Nicole Soranzo

**Affiliations:** 1 Department of Haematology, University of Cambridge, Cambridge Biomedical Campus, Long Road, Cambridge CB2 0PT, UK; 2 National Health Service (NHS) Blood and Transplant, Cambridge Biomedical Campus, Long Road, Cambridge CB2 0PT, UK; 3 Medical Research Council Biostatistics Unit, Cambridge Institute of Public Health, Cambridge Biomedical Campus, Forvie Site, Robinson Way, Cambridge CB2 0SR, UK; 4 MRC/BHF Cardiovascular Epidemiology Unit, Department of Public Health and Primary Care, University of Cambridge, Strangeways Research Laboratory, Wort’s Causeway, Cambridge CB1 8RN, UK; 5 Department of Human Genetics, The Wellcome Trust Sanger Institute, Wellcome Trust Genome Campus, Hinxton, Cambridge CB10 1HH, UK; 6 The National Institute for Health Research Blood and Transplant Unit (NIHR BTRU) in Donor Health and Genomics at the University of Cambridge, University of Cambridge, Strangeways Research Laboratory, Wort’s Causeway, Cambridge CB1 8RN, UK; 7 Blood Research Group, NHS Blood and Transplant, John Radcliffe Hospital, Headley Way, Headington, Oxford OX3 9BQ, UK; 8 European Molecular Biology Laboratory, European Bioinformatics Institute, Wellcome Trust Genome Campus, Hinxton, Cambridge CB10 1SD, UK; 9 Department of Haematology, Barts Health NHS Trust, The Royal London Hospital, Whitechapel Road, London, London E1 1BB, UK; 10 Department of Molecular Biology, Radboud University, Faculty of Science, Nijmegen 6525GA, the Netherlands; 11 Department of Medicine, University of Cambridge, Cambridge Biomedical Campus, Long Road, Cambridge CB2 0QQ, UK; 12 National Institute for Health Research Cambridge Biomedical Research Centre, Cambridge University Hospitals, Cambridge CB2 0QQ, UK; 13 NIHR BioResource-Rare Diseases, University of Cambridge, Cambridge Biomedical Campus, Long Road, Cambridge CB2 0PT, UK; 14 Département de Génétique et Développement (GEDEV), University of Geneva, 1211 Geneve 4, Switzerland; 15 Department of Cardiovascular Sciences, Center for Molecular and Vascular Biology, University of Leuven, 3000 Leuven, Belgium; 16 Wellcome Trust Centre for Human Genetics, University of Oxford, Roosevelt Drive, Oxford OX3 7BN, UK; 17 Department of Statistics, University of Oxford, 1 South Parks Road, Oxford OX1 3TG, UK; 18 UK Biobank Ltd., 1-4 Spectrum Way, Adswood, Stockport SK3 0SA, UK; 19 British Heart Foundation Centre of Excellence, Division of Cardiovascular Medicine, Addenbrooke’s Hospital, Hills Road, Cambridge CB2 0QQ, UK; 20 Emma Children’s Hospital, Academic Medical Center (AMC), University of Amsterdam, Location H7-230, Meibergdreef 9, Amsterdam 1105AZ, the Netherlands; 21 Blood Cell Research, Sanquin Research and Landsteiner Laboratory, Plesmanlaan 125, Amsterdam, 1066CX, the Netherlands; 22 Structural Biology and BioComputing Programme, Spanish National Cancer Research Centre (CNIO), Melchor Fernández Almagro, 3, 28029 Madrid, Spain; 23 Institute of Cellular Medicine, Newcastle University, Framlington Place, Newcastle upon Tyne NE2 4HH, UK; 24 Human Genetics, McGill University, 740 Dr. Penfield, Montreal, QC H3A 0G1, Canada; 25 Bioinformatics and Genomics, Centre for Genomic Regulation (CRG), Barcelona Institute of Science and Technology, Carrer del Dr. Aiguader, 88, Barcelona 8003, Spain; 26 Department of Experimental and Health Sciences, Universitat Pompeu Fabra (UPF), Plaça de la Mercè, 10- 12, Barcelona 8002, Spain; 27 Computational Genomics, Institut Hospital del Mar d’Investigacions Mediques (IMIM), Carrer del Dr. Aiguader, 88, Barcelona 8003, Spain; 28 UCL Cancer Institute, University College London, 72 Huntley Street, London WC1E 6BT, UK; 29 Radcliffe Department of Medicine, University of Oxford, John Radcliffe Hospital, Headley Way, Headington, Oxford OX3 9DU, UK; 30 Department of Haematology, Churchill Hospital, Headington, Oxford OX3 7LE, UK

**Keywords:** blood, genetics, hematology, epigenetics, hematopoiesis, Mendelian randomization, complex disease, autoimmune diseases, cardiovascular diseases

## Abstract

Many common variants have been associated with hematological traits, but identification of causal genes and pathways has proven challenging. We performed a genome-wide association analysis in the UK Biobank and INTERVAL studies, testing 29.5 million genetic variants for association with 36 red cell, white cell, and platelet properties in 173,480 European-ancestry participants. This effort yielded hundreds of low frequency (<5%) and rare (<1%) variants with a strong impact on blood cell phenotypes. Our data highlight general properties of the allelic architecture of complex traits, including the proportion of the heritable component of each blood trait explained by the polygenic signal across different genome regulatory domains. Finally, through Mendelian randomization, we provide evidence of shared genetic pathways linking blood cell indices with complex pathologies, including autoimmune diseases, schizophrenia, and coronary heart disease and evidence suggesting previously reported population associations between blood cell indices and cardiovascular disease may be non-causal.

## Highlights

• Genome-wide association study interrogates 36 traits across the hematopoietic system
• A total of 2,706 associated variants, including 130 rare and 230 low frequency
• Describes allelic spectrum and heritability of coding and regulatory variants
• Unravels causal contributions to cardiovascular, immune, and psychiatric disease

## Graphical Abstract

**Figure:**
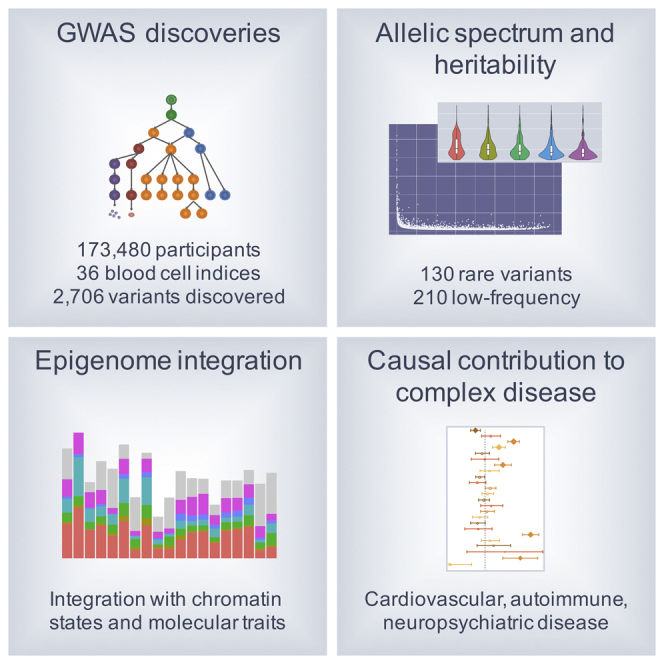

## Teaser

As part of the IHEC Consortium, this study probes the allelic architecture and regulatory landscape of cellular complex traits with power to identify causal pathways and links to diseases such as schizophrenia. Explore the *Cell Press* IHEC web portal at http://www.cell.com/consortium/IHEC.

## Introduction

Modern genetic analysis has transformed our understanding of the contribution of inherited variation to complex human disease. Over the last decade, the widespread application of large-scale genome-wide association studies based on sparse genotyping arrays has led to a dramatic increase in the number of known disease-associated genetic variants (Hindorff et al., 2009). The development of clinically useful applications of these discoveries, such as disease prediction algorithms, identification of etiological mechanisms (Ferreira et al., 2013, Voight et al., 2012), and prioritization of new targets for drug discovery (Lopez, 2008) has lagged behind. This is due partly to the characteristics of the disease-associated variants, which are predominantly common (minor allele frequency [MAF] ≥5%), which tend to be associated with small differences in disease risk and which often lie in regulatory regions of the genome, hindering the identification of causal alleles, genes, and disease mechanisms.

Examples of low-frequency (MAF = 1%–5%) and rare variant (MAF <1%) associations are beginning to emerge from the application of massively parallel whole genome and exome sequencing to human populations (Polfus et al., 2016). Associated rare variants tend to be easier to link to genes as they map predominantly in or near coding regions and have fewer correlated variants. Furthermore, they can have larger phenotypic effect sizes and are more likely to act through interpretable mechanisms such as disruption of protein function. These features also enhance their clinical and scientific usefulness. For instance, rare loss of function alleles can be used to assess the likely consequences of modulating a pathway pharmacologically to prevent disease (Plenge et al., 2013). However, very large studies are required for power to detect rare variant associations and consequently the sequencing approach is still relatively limited by cost.

Genotype imputation of large population cohorts (i.e., the systematic genome-wide statistical inference of unmeasured genotypes using exogenous reference panels of sequenced individuals) (Howie et al., 2011) is fast becoming a viable strategy to explore rare and low-frequency variant associations. Increasingly large whole-genome sequencing (WGS) reference panels are being created. Larger panels include rare alleles from more variants and better capture the between-variant correlation structure of study populations (1000 Genomes Project Consortium et al., 2015, Iotchkova et al., 2016b, Loh et al., 2016, UK10K Consortium et al., 2015). Here, we exploit the recent improvements in the quality of imputation to carry out association analyses of rare and low-frequency genetic variants with 36 different blood cell indices.

Blood cells make essential contributions to oxygen transport, hemostasis, and innate and acquired immune responses (Jenne et al., 2013, Jensen, 2009, Varol et al., 2015) and participate in many other functions such as iron homeostasis, the clearance of apoptotic cells and toxins, vascular and endothelial cell function, and response to systemic stress (Buttari et al., 2015). Qualitative or quantitative abnormalities of blood cell formation, and of their physiological and functional properties, have been associated with predisposition to cancer and with many severe congenital disorders including anemias, bleeding, and thrombotic disorders and immunodeficiencies (Routes et al., 2014, Schneider et al., 2015). Furthermore, variations in the properties of many blood-cell subtypes have been associated with a wide variety of systemic diseases. However, the causal relationships between blood indices and disease risks are unclear and this hinders their potential value for informing new treatments.

We report over 2,500 variants independently associated with variation in the 36 indices. We examine the genetic architecture of the associated variants and use them to reveal causal relationships with autoimmune, cardiovascular, and psychiatric diseases. Overall, this study expands the repertoire of genes and regulatory mechanisms governing hematopoietic development in humans and opens potential avenues for targeting key pathways involved in abnormal or dysregulated hematopoiesis.

## Results

### Genetic Discoveries

**Figure 1:**
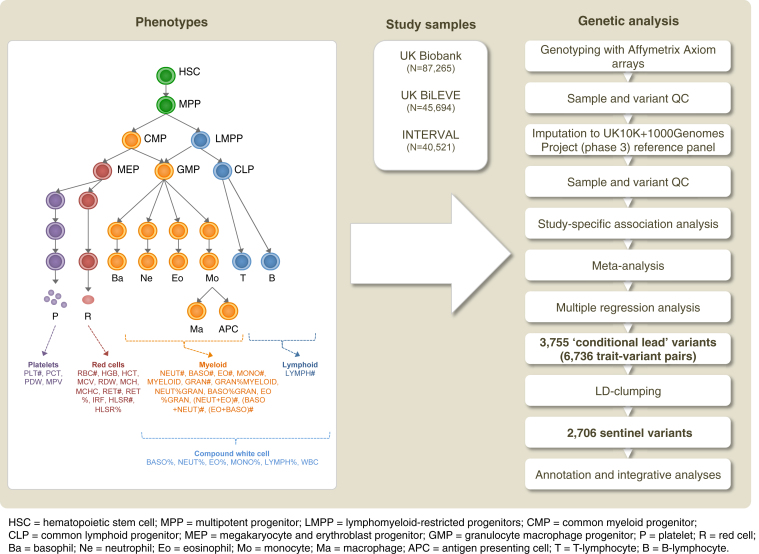
Study Design for GWAS of Complete Blood Count Indices The phenotypes and their classification by hematopoietic cell type; the study sample sizes; and a summary of the analysis methods employed to identify associated loci. Blood cell index names are defined in Table S1. See also Figures S1 and S2.

**Figure 2:**
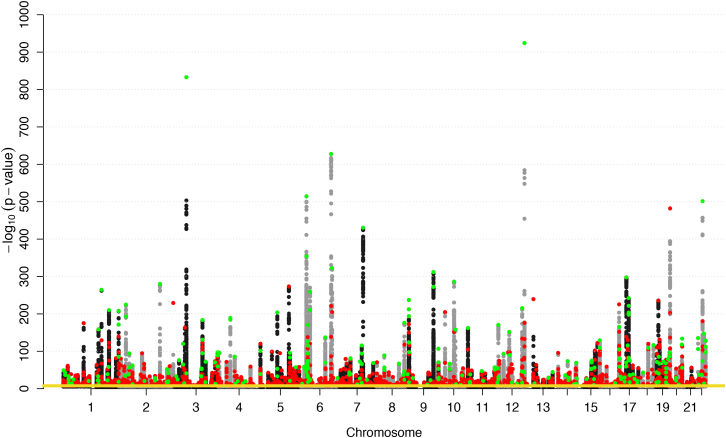
Summary of Genetic Associations with the 36 Blood Cell Indices A Manhattan plot summarizing genome-wide phenotypic associations over 36 indices. Each dot corresponds to a variant. Its x coordinate represents its genomic position and its y coordinate represents the maximum -log_10_ (p value) for association over all phenotypes. Variants with -log_10_ (p value) <6 have been removed for clarity. The yellow horizontal line at p = 8.31 × 10^−9^ represents the GWAS significance threshold. Sentinel variants are colored green if their associations (or associations with their proxies) have been previously reported and are colored red otherwise. See also Table S3.

**Figure S1:**
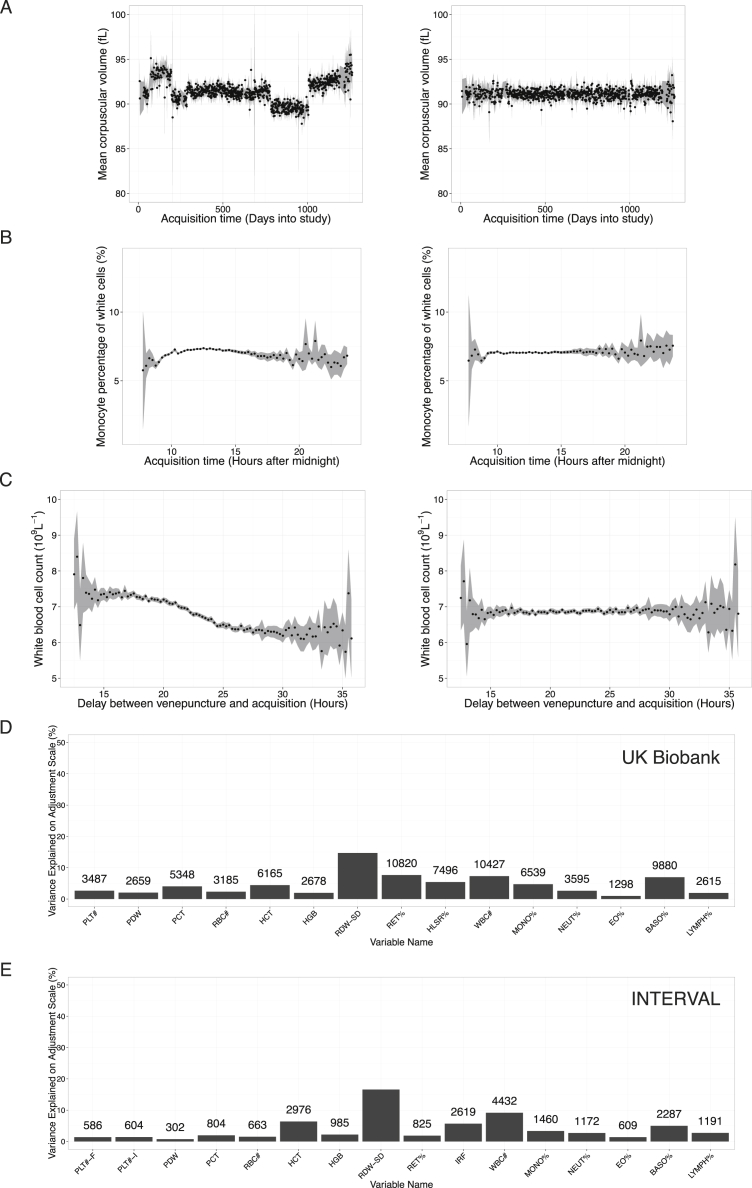
Adjustment for Technical Covariates Affecting Full Blood Count Measurements, Related to Figure 1, Tables S1 and S2, and the STAR Methods (A) Day averaged measurements of MCV taken from a single instrument over the course of UK Biobank baseline recruitment. The discontinuities may have been generated by calibration of the machine against a variable deterministically related to MCV. Continuous drift is visible within some of the piecewise continuous segments. The left plot is obtained using the raw data while the right plot is obtained using the technically adjusted trait, showing elimination of discontinuities and drift. (B) The effect of the time of day of acquisition on the average measurement of MONO%. Data are taken from a single Coulter instrument over the full UK Biobank baseline recruitment period. The left plot is obtained using the raw data while the right plot is obtained using the technically adjusted trait, showing elimination of the dependence of the mean of MONO% on time of day. (C) Example of the effect of time delay between venipuncture and acquisition on the measurement of the mean white blood cell count. Each point gives the average WBC# for samples acquired during baseline UK Biobank recruitment on a single Coulter instrument during a fifteen minute delay interval. The boundaries of the shaded region interpolate the 95% confidence intervals of the means. The left plot is obtained using the raw data while the right plot is obtained using the WBC# trait data that has been adjusted for the technical covariates. The dependence of the mean cell count on delay time has been eliminated. (D) Percentages of the variance of each UK Biobank measured variable explained by the adjustment for technical covariates and seasonal drift on the relevant adjustment scale. Integer labels show the effective number of additional samples gained from making the technical adjustments, meaning the expected number of additional samples that would be required to obtain equivalent p values in a GWAS for the trait if the adjustment were not made. (E) As for (D) except for INTERVAL.

**Figure S2:**
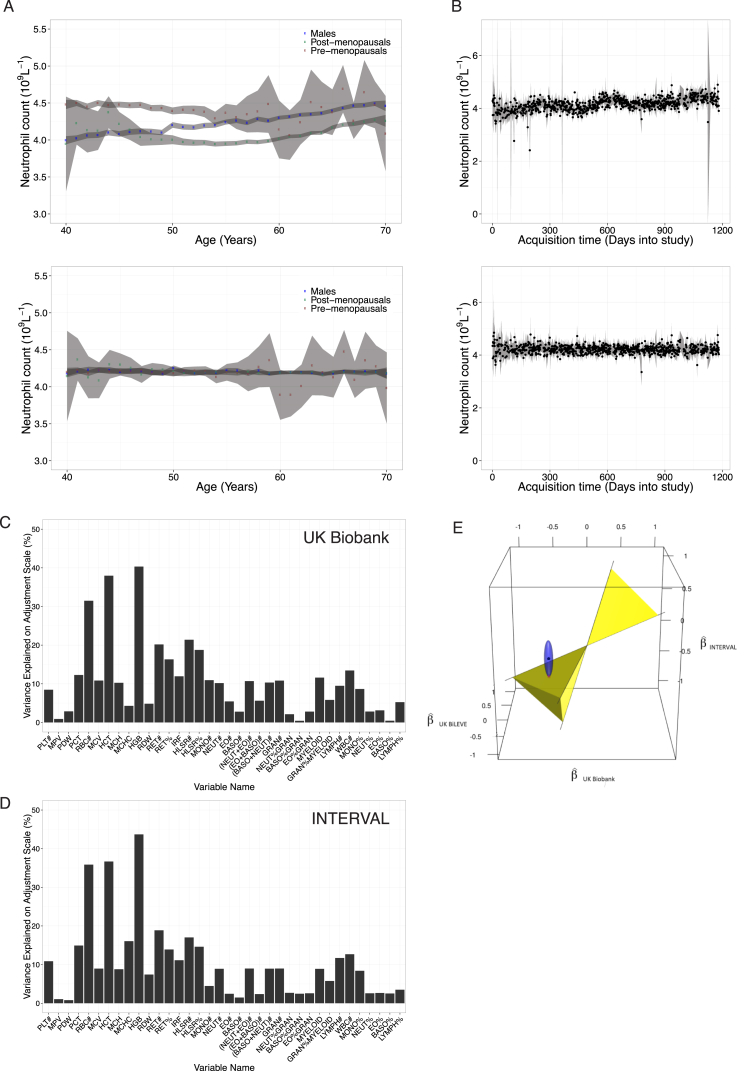
Adjustments for Sex and for Biological and Environmental Covariates Affecting Full Blood Count Measurements, Related to Figure 1, Tables S1 and S2, and the STAR Methods (A) The dependence of mean neutrophil count on sex and menopause status in the UK Biobank data adjusted for technical effects. The top plot is obtained using the raw data while the bottom plot is obtained adjusting the data for menopause and sex effects showing the elimination of the variance these covariates explain. (B) Day averaged measurements of neutrophil count taken from a single instrument over the course of the UK Biobank baseline recruitment. There is a long run upward drift in the average count over time. Seasonal oscillation in the average counts is also visible. The top plot is obtained using the raw data while the bottom plot is obtained using the technically adjusted data, showing the elimination of drift and seasonal oscillation. (C) Percentage of variance of UK Biobank traits explained (on the relevant adjustment scale) by sex and covariates affecting full blood counts, including age, menopausal status, smoking and alcohol variables. (D) As for (C) except for INTERVAL traits. (E) Illustration of the method used to determine the weight of evidence that heterogeneity in effect sizes across the three studies exceeded a tolerance criterion. The axes represent effect sizes in UK Biobank, INTERVAL and UK BiLEVE. The black dot represents the vector of study specific effect size estimates ($\hat{\beta }$_UK Biobank,_$\hat{\beta }$_INTERVAL_, $\hat{\beta }$_UK BiLEVE,_) for a variant. If the dot lies inside the infinite yellow double-pyramid (defined by three planes intersecting the origin, each normal to one of n_1_ = (1,−1/4, −1/4), n_2_ = (−1/4,1, −1/4), n_3_ = (−1/4,−1/4, 1)) we consider that there is no evidence of between study heterogeneity. If the black dot lies outside the yellow double-pyramid we measure the strength of evidence for heterogeneity as the distance between the black dot and the nearest point on the surface of the pyramid (red dot), with distances scaled to account for the standard errors of the study specific estimators. The nearest point on the pyramid is thus defined as the point in the smallest confidence surface for the estimators that intersects the pyramid (blue ellipsoid). We thresholded the distance score at 5.2 and filtered all variant-blood index pairs exceeding the score from further analysis.

**Figure S3:**
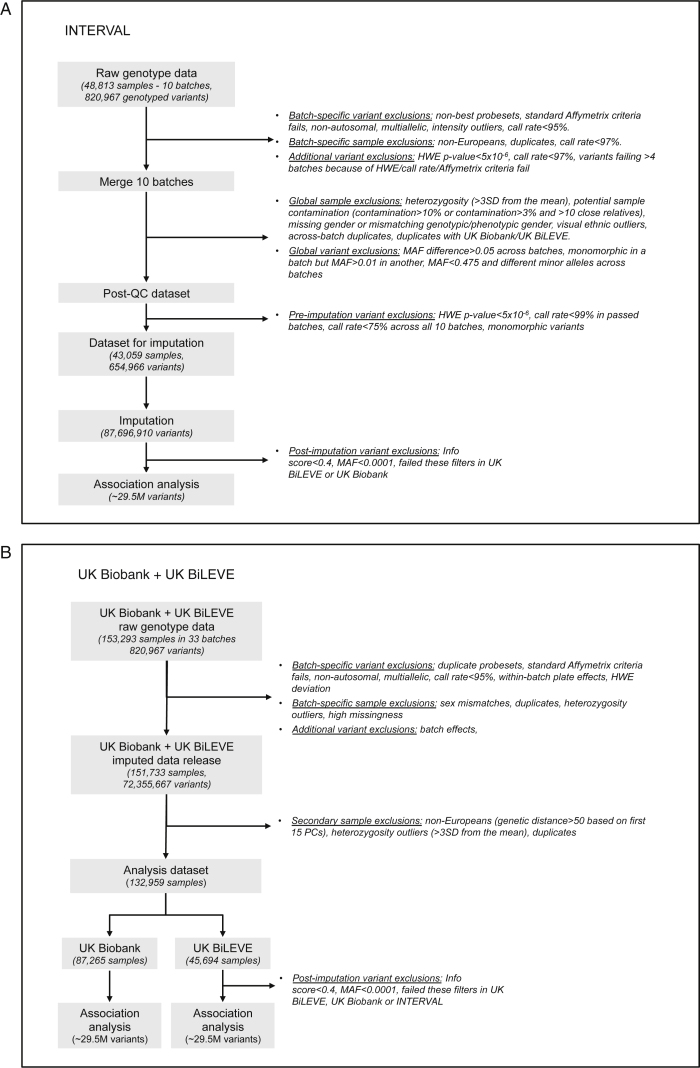
Quality Control of Genetic Data for UK Biobank, UK BiLEVE, and INTERVAL, Related to the STAR Methods Workflow describing QC steps for genotypic datasets. Detailed description of QC can be found in the STAR Methods and on the UK Biobank website (http://biobank.ctsu.ox.ac.uk/crystal/refer.cgi?id=155580). (A) INTERVAL samples. (B) UK Biobank + UK BiLEVE samples.

To identify genetic variants associated with 36 blood cell indices with increased resolution and statistical power, we studied a total of 173,480 European ancestry individuals from three large-scale UK studies—INTERVAL (Moore et al., 2014), approved by Cambridge (East) Research Ethics Committee, UK Biobank (Sudlow et al., 2015), and UK BiLEVE (a selected subset of the UK Biobank cohort) (Wain et al., 2015), both approved by the North West Multi-centre Research Ethics Committee (Figures 1, S1, and S2; Tables S1 and S2). We tested univariate associations of 36 indices with 29.5 million imputed variants passing quality control filters (MAF >0.01%, Figure S3) and used stepwise multiple regression to identify a parsimonious subset of genetic variants explaining the genome-wide significant (p value < 8.31 × 10^−9^) associations for each trait (Xu et al., 2014) (STAR Methods). We identified 6,736 conditionally independent index-variant associations and clustered these variants into 2,706 high linkage disequilibrium (LD) groups each represented by a sentinel variant (between-sentinel pairwise LD r^2^ < 0.8) (Figure 2; Tables S3 and S4). We confirmed the accurate imputation of variants at the rare end of the allelic spectrum by genotype comparisons with high read-depth (>50×) whole exome sequencing data from overlapping individuals, which showed 92.95% concordance and 94.97% precision for rare alleles (STAR Methods). Of the sentinel variants, 283 were correlated (r^2^ ≥ 0.8) with previously reported variants (Table S5), validating most blood trait associations reported in populations of European ancestry (Gieger et al., 2011, van der Harst et al., 2012, Vasquez et al., 2016).

**Figure 3:**
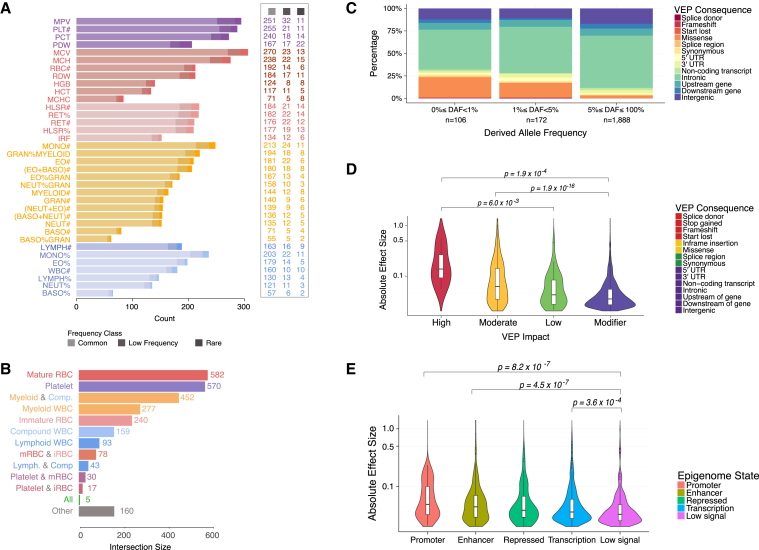
Distribution of Genetic Effects and Variant Consequences (A) Number of conditionally independent genetic associations categorized by blood cell index and by MAF range. (B) Summary of sizes of subsets of sentinel variants categorized by cell types of associated indices, showing that most associations are cell-type-specific. Each bar counts the number of sentinel variants associated with and only with the blood index class(es) shown. (mRBC, Mature RBC; iRBC, Immature RBC; Lymph, Lymphoid WBC; Comp, Compound WBC; All, Intersection of all blood index classes. “Other” counts variants uncounted by the other bars.) See Table S1 for blood index classification. (C) Bar plot showing the proportions of variants categorized by VEP consequence stratified by derived allele frequency (DAF) range. (D and E) Violin plots showing the distribution of the absolute value of the estimated effect size stratified by VEP impact categories (D) or cell-matched chromatin segmentation states (E). p values correspond to Mann-Whitney-Wilcoxon tests comparing the distributions indicated. See also Table S4.

The sentinel variants included an unprecedented number of low-frequency (n = 210) and rare (n = 130) alleles (Figure 3A). The genetic associations were almost completely cell-type-specific (Figure 3B), with 900 sentinels (33%) associated exclusively with red blood cell traits, 1,040 (38%) exclusively with white cell traits, and 570 (21%) exclusively with platelet traits. Only five common variants (at *ZFP36L2/THADA*, *SH2B3*, *HBS1L*, *PRTFDC1*, and *GCKR*) were associated with traits across all six trait classes defined in Table S1.

### Properties and Biological Significance of Associated Variants

To evaluate the representation of classes of genetic variants across the allele frequency spectrum, we annotated variants with their most severe consequence on GENCODE transcripts using VEP (McLaren et al., 2016). Variants predicted to have severe consequences (missense, frameshift, stop gained, start lost variants; Table S4) were highly enriched in the rare and low-frequency ranges, consistent with observations from large-scale sequencing projects (UK10K Consortium et al., 2015) and negative selection against variants affecting protein function (Figure 3C). Phenotypic effect sizes (the absolute additive change in trait mean measured in SD per allele) decreased with decreasing severity of the variant consequence (p = 2.2 × 10^−16^, Jonckheere-Terpstra test for trend in absolute value of effect size with VEP impact; Figure 3D). For instance, missense changes were overrepresented in the rare frequency range (p = 9.8 × 10^−29^, Pearson’s χ^2^ test) and displayed larger absolute effect sizes compared to non-missense variants (median 0.063 SD versus 0.035 SD, p = 2.5 × 10^−16^, Mann-Whitney-Wilcoxon test). There were also significant differences in median phenotypic effect sizes between variants mapping to five distinct regulatory states inferred from genome segmentations based on six histone marks in matched cells. Variants mapping to enhancer and promoter regions had larger median effect sizes than those mapping to other regulatory classes (Figure 3E).

Curated genes known to cause rare inherited Mendelian blood disorders (Greene et al., 2016, Westbury et al., 2015) were enriched among genes containing conditionally significant associations between variants altering protein sequence (missense, frameshift, stop gained, start lost variants) and blood indices of cell types matched to the disorders. For instance, we detected a 21.3 (95% confidence interval [CI]: 5.8–52.0) fold enrichment (FE) of Mendelian genes for bleeding, thrombotic and platelet disorders in the platelet-associated genes, a 34.0 FE (95% CI: 11.4–72.1) of genes carrying mutations for Mendelian diseases of the red blood cells in red cell genes and a 6.8 FE (95% CI: 2.2–15.6) of Mendelian genes for primary immune disorders in myeloid white cell genes. The enrichment overlaps included a known pathogenic missense variant (Landrum et al., 2016) in myeloperoxidase deficiency (*MPO*) (Romano et al., 1997), and we identified additional known pathogenic variants in uncurated genes including *CX3CR1* (HIV progression) (Faure et al., 2000) and hemochromatosis type 1 (*HFE*) (Adams et al., 2005) (Table S4). We also found rare missense variants in Mendelian disorder genes that had not previously been associated with blood cell indices (Table S3) and/or where no pathogenic variants have been recorded in ClinVar. For example, missense variants in *GMPR*, *TMC8*, and *RIOK3* were associated with reticulocyte counts.

More generally, the 158 variants predicted to alter protein sequence (Table S4) are of interest because of their potential medical value. We focused on rare (MAF < 1%) protein-altering variants because they can be more reliably linked to causal genes. For red blood cell indices, we found 14 missense variants and one frameshift variant (in *SPHK1*), only one of which (rs116100695) was previously identified as pathogenic. rs116100695 is a rare missense variant in *PKLR* causing red cell pyruvate kinase deficiency, a common cause of hereditary nonspherocytic hemolytic anemia (Kanno and Miwa, 1991). Some of the other variants are in genes previously associated with hereditary anemias. For example, a rare missense variant (rs201514157) in *SPTA1* was associated with reticulocyte count, and a rare missense variant (rs202099525) in *PIEZO1* was associated with mean corpuscular hemoglobin concentration. Similarly we identified 11 rare protein-altering variants associated with platelet indices, ten of which were missense variants and one a nonsense variant (in *KALRN*). These include variants from regions previously identified to contain common weak-effect variants (*IQGAP2*, *JAK2*, *SH2B3*, and *TUBB1*) but also from three gene regions not previously identified by GWAS (*CKAP2L*, *PLEK*, and *TNFRSF13B*).

We identified 11 rare protein-altering variants associated with white cell traits, including ten missense variants in regions previously associated (*CEBPE*, *CXCR2*, *IL17RA*, *S1PR4*), as well as in novel genes not previously known to play a role in hematological processes. These findings demonstrate roles in leukocyte formation and/or function for *ALOX15*, *AMICA1*, and *PLEK*. Finally, some rare missense variants had pleiotropic effects across cell types. For instance, the rare missense variant in *TNFRSF13B* (rs72553883) causing common variable immunodeficiency and selective immunoglobulin A deficiency (Castigli et al., 2005) was associated with platelet, myeloid white cell and lymphoid white cell indices (Table S4).

Overall, these results expand our knowledge of the genes and regulatory regions controlling blood cell biology and function. For rare variants, there were too few minor allele homozygotes to estimate precisely genotypic effects on phenotype, even across >170,000 individuals. However, the magnitude of some rare heterozygote effects suggests that the corresponding homozygote effects could be clinically relevant. Indeed, it is possible that effects of some homozygotes are more than double those of corresponding heterozygotes depending on the degree of loss or gain of function, possible compensatory pathways, and stress or demand for adaptation in response to injury or insult.

### Allelic Architecture of Hematological Indices

**Figure 4:**
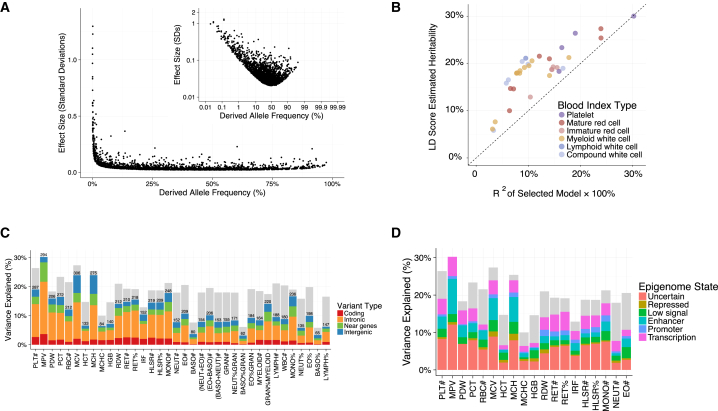
Allelic Architecture of Blood Cell Indices (A) Scatterplot showing the relationship between estimated derived allele frequency (DAF) and the absolute value of the estimated effect size for the sentinel variants. The inset gives the same plot on the logit/log scales. Only associations annotated with an ancestral allele are shown. (B) Scatterplot of LD score estimated heritability (due to common variants) against the (unadjusted) phenotypic variance explained by the conditionally significant variants in a multiple regression model, colored according to index type. (C) A barplot showing the LD score estimated heritability due to common variants (upper limit of gray bars) and the distribution of the unadjusted proportion of phenotypic variance explained (R^2^) by the conditionally significant variants grouped by genomic location (range of color fills). (D) The same plot for variants grouped by cell-matched chromatin segmentation states. See also Table S4.

The comprehensive nature of this study allows us to draw more general inferences about the allelic architecture of hematological indices as an exemplar class of complex human traits. Our analysis had at least 80% power to detect associations explaining 0.0265% of trait variance, which could be attained by a per-allele additive effect as small as 0.023 phenotypic SD for common (MAF ≥5%) variants and 1.154 SD for variants at the lower limit of the frequency range we considered (MAF = 0.01%). No common or low-frequency variant had an estimated absolute effect size >0.5 SD, suggesting an upper boundary on phenotypic effect sizes for variants in these frequency classes. The relationship between allele frequency and the absolute value of the estimated effect size for the sentinel variants could in principle be explained by differential winner’s curse by allele frequency (Figure 4A). However, the strength of the signal strongly suggests natural selection against variants with large effects. Conversely, associations with large phenotypic effects were overrepresented among rare variants (p value = 1.58 × 10^−77^, Pearson’s χ^2^ test), with 21 rare sentinel variants having an estimated effect size >0.5 SD (median MAF = 0.09%), five of which had effects greater than 1 SD (Table S4). These correspond to effects on traits of 2.73 g/dl, 3.77 fL (femtoliters), 51 × 10^9^/L, and 1.37 × 10^9^/L for hemoglobin concentration (HGB), mean corpuscular volume (MCV), and platelet and neutrophil counts, respectively. The effect sizes seen in heterozygotes are sufficiently large to cause disease when carried in homozygosis.

Using the LD score regression (Finucane et al., 2015) approach to polygenic modeling, we estimated that common autosomal genotypes explained between 18% and 30% of variance in platelet indices, between 10% and 28% of variance in red cell indices, and between 5% and 21% of variance in white cell indices (Figure 4B). Conditionally significant coding variants explained between 0.2% and 3.7% of trait variance (R^2^ unadjusted for winner’s curse), while intronic variants, variants near genes, and intergenic variants explained between 1.2% and 18.0%, between 0.6% and 6.7%, and between 0.5% and 6.4% of trait variance, respectively (Figure 4C). Interestingly, conditionally significant variants associated with mean platelet volume (MPV) explain a slightly larger proportion of trait variance than the polygenic common-variant estimate of heritability made by LD score. This suggests that the low frequency and rare variants we discovered contribute more to heritability than the undiscovered common variants. The extent of the winner’s curse effect will need to be assessed when comparable datasets become available (e.g., the remaining ∼350,000 UK Biobank participants), but if the effect is weak, we may have identified almost all common variants with non-negligible effects associated with MPV. However, as a substantial proportion of the common-variant heritable variance of most blood cell indices remains unexplained by the conditionally significant genetic variants, it is likely that many more common variants of small effect remain to be discovered. Moreover, larger studies are also likely to identify even rarer variants with stronger effects, which will be clinically valuable.

**Figure 5:**
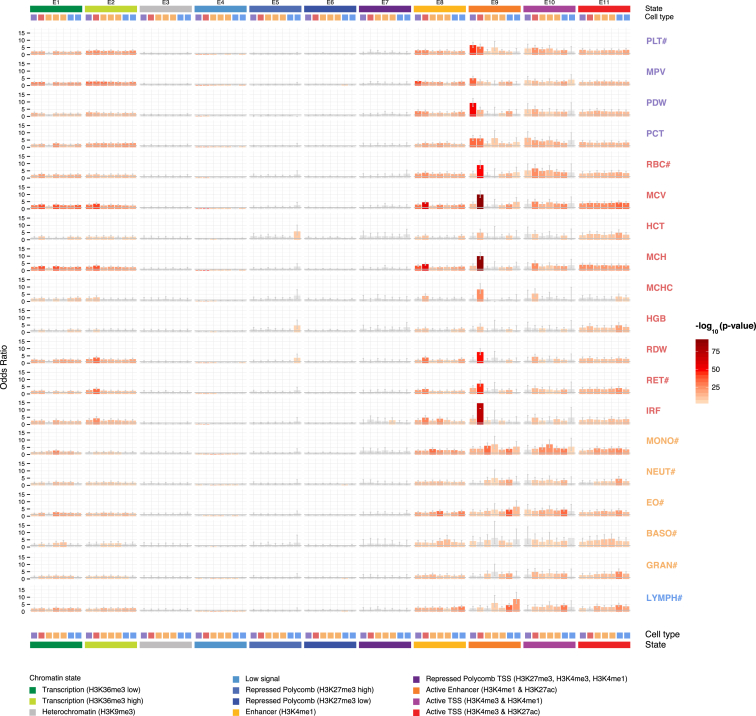
Enrichment of Trait Associations within Regulatory Regions Odds ratios (bar heights) and 95% confidence intervals (whiskers) for enrichment of blood-index associations with chromatin segmentation states from blood cells. P values for significance are obtained from a generalized linear model, modeling a threshold on the GWAS test statistic as a Bernoulli response while controlling for MAF, distance from gene, and number of LD proxies. The cell types are shown from left to right in each block as follows: megakaryocyte (i.e., the platelet progenitor, purple), erythroblast (i.e., the red cell progenitor, red), monocyte (orange), eosinophil (orange), neutrophil (orange), naive B cell (light blue), and T cell (light blue). See also Table S4.

Finally, we estimated the proportion of the heritable component of each blood cell index that was explained by the polygenic signal across different genome regulatory domains, as defined by chromatin segmentation states in the relevant cell types (Carrillo de Santa Pau et al., 2016). We found that variants lying in enhancers explained 19%–46% of heritable variation, with similar estimates for transcribed regions (15%–48%), and lower estimates for promoters (4%–30%) and silencers (3%–15%). Additionally, we estimated the variance explained by the conditionally significant variants using multiple regression, showing that the identified signals are distributed across regulatory states (Figure 4D). To understand the extent to which these patterns may be driven by cell-type-specific regulatory elements, we used a robust non-parametric analysis approach to test the significance of enrichments of each set of summary statistics against cell-type matched and cell-type discordant chromatin segmentation states (Iotchkova et al., 2016a). Active enhancer regions defined by H3K4me1/H3K27ac histone modifications (E9, Figure 5) demonstrated striking patterns of cell-type specificity of enrichments compared to those defined by other chromatin states. For example, we saw up to 15-fold enrichment of red-cell associations in corresponding active enhancer regions and up to 10-fold enrichment for platelet signals in megakaryocyte (the platelet progenitor cell) enhancers. There was also statistically significant evidence for depletion of associated variants in transcriptionally inactive regions.

### Regulatory Consequences of Blood-Cell-Associated Variants

The linking of regulatory variants to their effector genes and mechanisms continues to be a challenge for the complex traits community. Public resources that annotate sequence variation facilitate the task through overlap with molecular traits including cell-type-specific chromatin states and transcription factors (Roadmap Epigenomics Consortium et al., 2015), gene expression quantitative trait locus datasets (eQTL) (GTEx Consortium, 2015), or, more recently, annotation of physical interactions between different regions of the genome (Hughes et al., 2014). However, as the fraction of the genome that is annotated continues to increase, so does the risk of non-functional (random) overlap. The intersection of genetic and regulatory data at the level of individual genetic variants allows formal modeling of the probability that a cellular or organismal trait “colocalizes” with its molecular counterpart, thus allowing the robust assignment of candidate genes and functional mechanisms to GWAS variants. For example, in a companion paper by the BLUEPRINT project, we have shown that only ∼25% of disease associations that were in high LD (r^2^ ≥ 0.8) with a given molecular event had a high probability (>99%) of colocalization (Chen et al., 2016).

**Figure 6:**
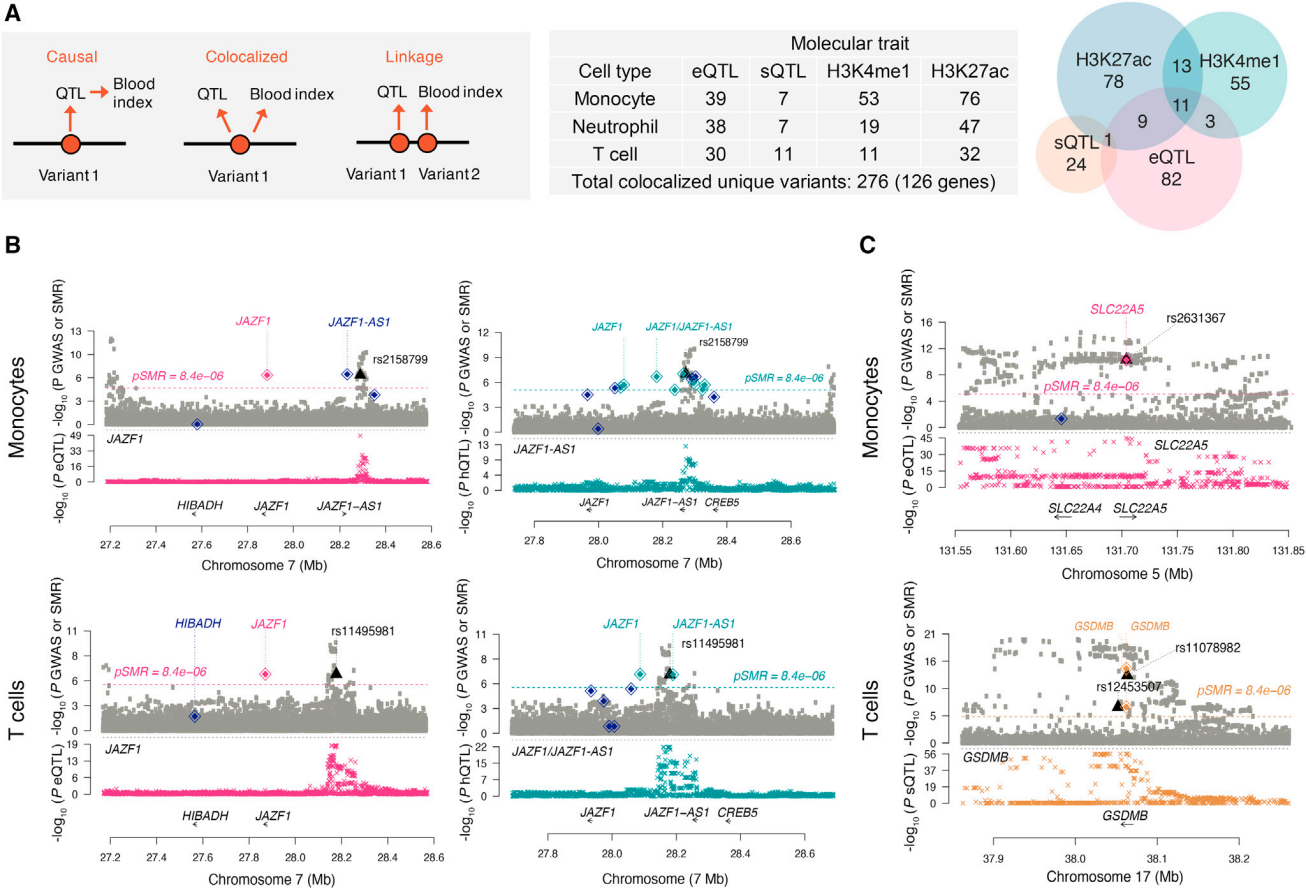
Colocalization between Cellular and Molecular Traits (A) Illustrates the models tested using SMR, as well as the number of variants that were significant for both the cellular and molecular trait at a p value threshold of 8.4 × 10^−6^ that show colocalization (P_HEIDI_ > 0.05) between the cellular and the molecular trait and the overlap of colocalized marks between the four marks across the three cell types. (B and C) Regional plots for the colocalization result in the (B) *JAZF1*, (C) *SLC22A5I* and *GSDMB* loci for monocytes and T cells. The gray squares represent the p value distribution for the corresponding (monocyte and lymphocyte) blood cell index. The black triangles represent the GWAS variant that colocalizes with the eQTL (pink diamond), hQTL (light blue diamonds), and sQTL (gold diamond). The dark blue diamonds represent QTL in the region that do not show colocalization. The crosses represent the regional QTL p value distribution. See also Table S6.

The Chen et al. (2016) dataset includes three primary human cell types (classical monocytes, neutrophils, and CD4^+^ naive T cells) matched to our blood indices. We thus accessed summary statistics generated for gene expression (eQTL), mRNA splicing (sQTL), and histone modifications marking enhancers and promoters (H3K4me1 hQTL and H3K27ac hQTL) and used summary-data-based Mendelian randomization (SMR) analysis (Zhu et al., 2016) to test for colocalization of signal between molecular and blood cell index GWAS in matched cell types (MONO#, NEUT#, and LYMPH#) (Figure 6A).

Across all the three cell-types and the four QTL datasets, there were 276 cell trait variants that colocalized with at least one molecular QTL in the corresponding cell type, indicating a shared genetic influence on the two phenotypes (p value _HEIDI_ > 0.05; Table S6). As in the Chen study, only ∼30% of overlapping associations detected resulted in a robust colocalization. Overall, we can thus assign a putative functional interpretation to ∼10% of all sentinel variants. Only 47% of colocalizing signals involved changes in gene expression or mRNA splicing (126 unique genes), indicating likely effector genes underpinning associations. These include disease-associated variants (e.g., an eQTL for *SLC22A5* associated with Crohn’s disease and a sQTL for *GSDMB* associated with a range of autoimmune diseases) (Figures 6B and 6C). The remaining 53% of signals involved histone modifications, indicating a regulatory change not associated with detectable expression changes in our data. Interestingly, 24 instances involving both gene expression and histone modifications at closely located variants allow us to assign putative regulatory elements to their effector genes, as illustrated by the case of the *JAZF1* locus (Figure 6B). Overall, these examples show how genetic variants affect cellular traits and complex disease through molecular mechanisms of gene regulation.

### Causal Contribution of Hematological Trait Variation to Common Complex Diseases

Patients with complex disease often display abnormal blood cell index levels, but it is not always clear whether these reflect etiological roles of hematological pathways or are a consequence of disease. As pharmacological modulation of blood cell indices advances, identifying shared causal pathways between these indices and complex diseases could provide new therapeutic opportunities. Mendelian randomization (MR) uses genetic variants to estimate causal associations, reducing the potential for confounding and reverse causation that limit observational studies.

**Figure 7:**
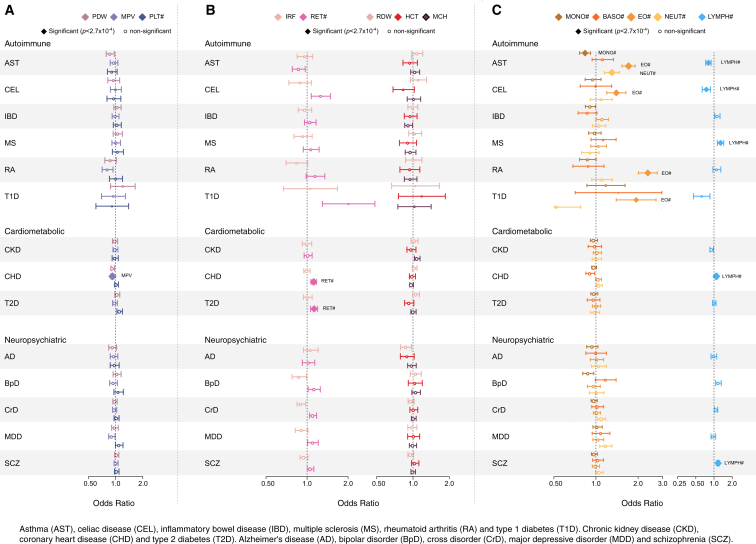
Causal Associations with Common Diseases (A–C) A forest plot showing the results of the multivariable Mendelian randomization (MR) analysis conducted on 13 blood cell indices versus fourteen common diseases. Colored diamonds represent the significant trait-disease association at our Bonferroni corrected p value threshold of 2.7 × 10^−4^ with uncolored circles denoting non-significant results. Each diamond/circle represents the estimated unconfounded causal odds ratio of disease risk per SD increase of the blood cell index, adjusted for all other blood cell indices tested. The size of the shape is inversely proportional to the SE and the whiskers denote 95% confidence intervals. Forest plots are presented for (A) platelet indices, (B) immature and mature red cell indices, and (C) myeloid and lymphoid white cell indices. See also Table S7.

We conducted a multivariable MR analysis to reassess epidemiological correlations between blood cell indices and a range of human complex diseases and to identify shared causal pathways. The multivariable approach is advantageous because it ensures that results for one index are conditional on (i.e., control for covariation in) all other indices. For this analysis, we retrieved publicly available summary statistics for six autoimmune, three cardiometabolic, and five neuropsychiatric diseases (STAR Methods) and used genetic variants associated with 13 main hematological indices. For each index-disease pair, we estimated the unconfounded increase in the odds ratio of disease per unit change (in SD) in the index. We applied a multiple testing correction for 182 disease-index comparisons (Figure 7).

We detected significant associations between white blood cell indices and autoimmune diseases (Figure 7C). The strongest was a positive association between eosinophilic indices and asthma (asthma odds ratio [OR] per SD increase in eosinophil count = 1.71; 95% CI: 1.53–1.95; p = 4.0 × 10^−22^). This finding corroborates evidence from known associations with eosinophil counts at confirmed asthma loci, such as *IL5*, *IL33*, and *IL1R1*, as well as our discovery that the region around *TSLP* (another known asthma locus) contains three independent signals associated with eosinophil count (Table S4). There was weaker evidence of a positive association between asthma and neutrophil indices (p = 2.74 × 10^−5^), as well as inverse associations with monocyte (p = 1.24 × 10^−4^) and lymphocyte (p = 7.56 × 10^−5^) counts. There was also strong evidence for a positive association between eosinophilic indices and rheumatoid arthritis (OR = 2.34, 95% CI: 2.01–2.74; p = 1.84 × 10^−27^), a signal that was robust to a range of sensitivity analyses, including removal of the MHC region (Table S7). Other loci containing alleles robustly associated with higher eosinophil count and increased risk of rheumatoid arthritis were *COG6*, *SPRED2*, *RUNX1*, and the highly pleiotropic *ATXN2/SH2B3/BRAP* region (Table S4).

As with eosinophils, we saw directionally discordant disease associations with lymphocyte count, which had positive associations with schizophrenia (OR = 1.17, 95% CI: 1.10-1.24; p = 1.1 × 10^−7^), multiple sclerosis (OR = 1.28, 95% CI: 1.14–1.45; p = 6.6 × 10^−5^), and coronary heart disease (CHD) (OR = 1.10, 95% CI: 1.04–1.15; p = 1.8 × 10^−4^), as well as inverse associations with asthma (OR = 0.81, 95% CI: 0.73–0.90; p = 7.6 × 10^−5^) and celiac disease (OR = 0.75, 95% CI: 0.64–0.87; p = 2.6 × 10^−4^). However, only the associations with multiple sclerosis and celiac disease were robust to removal of the MHC region, suggesting that genes within MHC predominantly drive the links between schizophrenia, coronary artery disease, and asthma. Finally, there was a weak positive association of CHD risk with reticulocyte indices (OR = 1.12; 95% CI: 1.07–1.17; p value = 1.7 × 10^−6^) and a weak inverse association of CHD risk with MPV (OR = 0.92; 95% CI: 0.88–0.96; p = 8.1 × 10^−5^), both of which were robust to all sensitivity analyses (Figure 7).

These analyses have suggested a weak but significant positive association between hemolysis and CHD risk. This may prompt re-evaluation of the risk of arterial thrombosis for patients with on-going hemolysis as has been done for venous thrombosis. Perhaps, most strikingly the association between eosinophil count and rheumatoid arthritis may trigger more detailed genetic and clinico-epidemiological studies to dissect the provoking and perpetuating pathology of this inflammatory disease.

## Discussion

The molecular programs that control hematopoietic stem cell differentiation and proliferation are incompletely understood (Notta et al., 2016, Paul et al., 2015). Clues to these molecular pathways have traditionally come from discoveries of highly penetrant mutations associated with inherited disorders of the hematopoietic system, somatic mutations underlying blood cell cancers, and from functional screens in model organisms (Boatman et al., 2013, Ganz and Nemeth, 2012). More recently, such studies have been complemented by high-throughput molecular and genetic analyses of common biological variation (Vasquez et al., 2016). Our study benefited from a substantial increase in statistical power compared to previous GWAS, driven by improvements in study design and data capture, including the use of dense WGS-imputation panels and the accurate adjustment of phenotypes for biological and technical covariates.

The new associations, including a large number of rare and low-frequency coding variants, define a detailed atlas of genes and regulatory regions influencing blood cell indices with cell-type-specific effects. There were several rare variants in genes known to carry mutations causing severe disorders. For example, rs149000560, a rare missense variant associated with immature red cell indices, lies in *FERMT3*, the gene responsible for the leukocyte adhesion deficiency-1/variant syndrome (Kuijpers et al., 2009). Loss of function mutations in *CKAP2L* associated with platelet traits cause the autosomal-recessive Filippi syndrome characterized by microcephaly, pre-, and post-natal growth failure, although case series do not describe hematological abnormalities (Hussain et al., 2014). *CKAP2L* is associated with microtubules in dividing cells and the association of a mutation in this gene with platelet phenotype and cortical development reflects the role of tubulin function in megakaryopoiesis and neuronal migration (Moon and Wynshaw-Boris, 2013). *PLEK* (encoding pleckstrin) is not known to carry rare pathogenetic mutations but it is a crucial protein for platelet function. Platelets from mice lacking pleckstrin exhibit a marked defect in exocytosis of delta and alpha granules, alphaIIbbeta3 activation, actin assembly, and aggregation (Lian et al., 2009). Other variants point to previously unknown genes. For instance, the biological functions of *TMC8* and *RIOK3* in developing erythroid cells are not understood but their associations with specific blood cell phenotypes may inform future experimental studies. For example, *RIOK3* has been associated with organization of the actin cytoskeleton, as a component of pre-40S pre-ribosomal particle and as mediating phosphorylation of MDA5.

Finally, other rare variants were potentially regulatory, mapping to intronic regions of genes not expressed in the relevant cell types. For instance, there was a series of intronic variants associated with MCV in *NPRL3*, *LUC7L*, *ITFG3*, and *AXIN1* genes that lie within 1Mb of the alpha globin gene. Such variants may be in LD with a deletion of the respective variants of alpha globin (HGA), but it is also possible that the respective variants are disrupting long-range enhancers of alpha-globin.

One intriguing set of associations with multiple hematopoietic lineages was of variants in genes involved in sphingosine signaling. A frameshift variant in the sphingosine-1-phosphate kinase gene (*S1PK*) and a missense variant in the sphingosine-1-phosphate receptor gene (*S1PR2*), which is expressed during erythroid development, were associated with altered reticulocyte count. Missense alleles in *S1PR4* are associated with reduced neutrophil, monocyte, and eosinophil counts consistent with previous reports (CHARGE Consortium Hematology Working Group, 2016). Taken together, these data suggest sphingosine-1-phosphate may be involved with the release and/or survival of red cells as well as white cells.

Variation in blood cell indices has been linked to diseases with high population burdens, including chronic complex conditions such as autoimmune disease, susceptibility to infection, and respiratory and cardiovascular illnesses. Here, we used Mendelian randomization inference to unravel causal mechanisms underlying reported index-disease correlations and applied a range of sensitivity analyses. Our genetic evidence for a causal role of eosinophilic pathways in asthma supports the pathophysiological and pharmacological evidence that eosinophils are key effector cells in asthma pathogenesis (Zijlmans et al., 2008). More surprising was the strong evidence for a positive association between eosinophilic indices and rheumatoid arthritis. Unexplained eosinophilia has been reported in rheumatoid patients, and the magnitude of eosinophilia has been associated with disease severity or activity, but little attention has been given to a pathogenetic role of eosinophils in rheumatoid arthritis. Our data support recent hypotheses linking eosinophil activation in rheumatoid processes (Rosenstein et al., 2014). Eosinophilic indices were also weakly positively associated with both celiac disease (p = 3.28 × 10^−5^) and type 1 diabetes (p = 7.66 × 10^−5^), highlighting a key role of eosinophils in pathways influencing the development of a range of autoimmune diseases.

Immune system dysfunction has been suspected to play a role in schizophrenia, a hypothesis supported by abnormal lymphocyte levels seen in schizophrenic patients but lacking support from longitudinal data (Miller et al., 2013). Our finding of shared genetic links between lymphocyte count and schizophrenia at the MHC region through multiple independent pathways may support a pathogenic role for immune dysfunction in development of schizophrenia, exemplified by the recent identification of key complement factor genes (*C4A*, *C4B*) as drivers of schizophrenia (Sekar et al., 2016). The positive association of lymphocyte count with multiple sclerosis is also confirmatory of the assumed pathogenetic role of T cells and is supported by the strong enrichment of genes involved in T cell activation or proliferation among known multiple sclerosis loci (Sawcer et al., 2011).

The most intriguing observations were the weak positive association of CHD risk with reticulocyte indices and the weak inverse association of CHD risk with MPV. Reticulocyte count and percentage are indicators of erythrocyte turnover and higher levels indicate increased hemolysis, which leads to increased levels of circulating free hemoglobin. Our data were consistent with previous studies that have shown that reduced clearance of free hemoglobin in carriers of the haptoglobin Hp2-2 allotype is associated with more oxidative stress and inflammation (Asleh et al., 2005, Kristiansen et al., 2001) and is associated with a higher risk of CHD events in type 1 diabetes (Ijäs et al., 2013, Levy et al., 2002). Moreover, it is also well established that free hemoglobin in blood substitutes leads to reduced nitrous oxide, increased vasoconstriction, and a higher risk of acute myocardial ischemia (Natanson et al., 2008). Our data support the hypothesis that hemolysis and risk of CHD are influenced by shared causal pathways. However, the pathways through which increased MPV could be protective of atherosclerotic disease remain to be determined, as does the apparent contradiction with prospective observational studies, which have reported associations in the opposite direction (Sansanayudh et al., 2014).

Finally, we were also able to reduce the likelihood of causality for several previously reported observational associations between blood cell indices and risks for various complex diseases, including previously reported associations of total white blood cell, granulocyte, and neutrophil counts with CHD risk (Wheeler et al., 2004) and type 2 diabetes (Gkrania-Klotsas et al., 2010), and red blood cell count associations with CHD risk (Schaffer et al., 2015) and red cell distribution width and mean corpuscular volume with type 2 diabetes (Engström et al., 2014). This suggests that the original observational studies were likely to be confounded.

In conclusion, the discovery of a substantial number of rare alleles with large effect sizes highlights the potential of large-scale population studies to identify variants on a continuum between extremely rare highly penetrant mutations driving Mendelian disorders and common variants of weak effect typically identified by GWAS. Our results are expected to boost current efforts to identify and assess possible novel etiologies and therapeutic targets for hematological diseases. Some of the variants discovered have phenotypic effects of large magnitude, perhaps sufficient to cause disease if carried in homozygosis. Carrier status may influence the interpretation of clinical tests of blood cell indices, and the variants and loci could be incorporated into the current diagnostic panels for inherited anemia and thrombocytopenia after biological validation of these results (Lentaigne et al., 2016, Roy et al., 2016).

## STAR★Methods

### Key Resources Table

**Table:** 

REAGENT or RESOURCE	SOURCE	IDENTIFIER
**Software and Algorithms**		

flashpca	(Abraham and Inouye, 2014)	https://github.com/gabraham/flashpca
R 3.1.2	(R Core Team, 2014)	https://www.r-project.org/
biomaRt	Bioconductor	https://bioconductor.org/packages/release/bioc/html/biomaRt.html
data.table	The R Foundation	https://cran.r-project.org/web/packages/data.table/index.html
doMC	The R Foundation	https://cran.r-project.org/web/packages/doMC/index.html
dplyr	The R Foundation	https://cran.r-project.org/web/packages/dplyr/index.html
foreach	The R Foundation	https://cran.r-project.org/web/packages/foreach/index.html
GenomicRanges	Bioconductor	https://bioconductor.org/packages/release/bioc/html/GenomicRanges.html
Hmisc	The R Foundation	https://cran.r-project.org/web/packages/Hmisc/index.html
openxlsx	The R Foundation	https://cran.r-project.org/web/packages/openxlsx/index.html
RcppEigen	The R Foundation	https://cran.r-project.org/web/packages/RcppEigen/index.html
reshape2	The R Foundation	https://cran.r-project.org/web/packages/reshape2/index.html
rhdf5	Bioconductor	https://www.bioconductor.org/packages/release/bioc/html/rhdf5.html
stringr	The R Foundation	https://cran.r-project.org/web/packages/stringr/index.html
tidyr	The R Foundation	https://cran.r-project.org/web/packages/tidyr/index.html
Hmisc	The R Foundation	https://cran.r-project.org/web/packages/Hmisc/index.html
MASS	The R Foundation	https://cran.r-project.org/web/packages/MASS/index.html
ggplot2	The R Foundation	https://cran.r-project.org/web/packages/ggplot2/index.html
lubridate	The R Foundation	https://cran.r-project.org/web/packages/lubridate/index.html
mgcv	The R Foundation	https://cran.r-project.org/web/packages/mgcv/index.html
RColorBrewer	The R Foundation	https://cran.r-project.org/web/packages/RColorBrewer/index.html
PLINK v1.9	(Chang et al., 2015)	https://www.cog-genomics.org/plink2
SHAPEIT3	(O’Connell et al., 2016)	https://jmarchini.org/software/
PBWT	(Durbin, 2014)	https://imputation.sanger.ac.uk/
BOLT-LMM	(Loh et al., 2015)	https://data.broadinstitute.org/alkesgroup/BOLT-LMM/
METAL	(Willer et al., 2010)	http://csg.sph.umich.edu//abecasis/Metal/
SMR	(Zhu et al., 2016)	http://cnsgenomics.com/software/smr/

**Other**		

Clinvar database	(Landrum et al., 2016)	https://www.ncbi.nlm.nih.gov/clinvar/
Variant Effect Predictor	(McLaren et al., 2016)	http://www.ensembl.org/info/docs/tools/vep/index.html
PhenoScanner	(Staley et al., 2016)	http://www.phenoscanner.medschl.cam.ac.uk/phenoscanner

### Contact for Reagent and Resource Sharing

Further information may be directed to the Lead Contact, Nicole Soranzo (ns6@sanger.ac.uk). Results, including genome-wide univariable summary statistics, are available from http://www.bloodcellgenetics.org.

### Experimental Model and Subject Details

We analyzed data from three large population studies with measurements of blood cell indices and imputed genome-wide genotypes - the UK Biobank study, the UK BiLEVE study (a selected subset of UK Biobank) and the INTERVAL study. Although the UK BiLEVE study is a subset of the UK Biobank study, we often refer to the UK BiLEVE study separately, since we conducted association analyses of UK BiLEVE participants as a distinct dataset due to their selected nature and a slightly different genotyping array.

#### The UK Biobank Study

The UK Biobank study is a prospective cohort study of 502,682 participants recruited at 22 assessment centers across the UK between 2006 and 2010 (Sudlow et al., 2015). Participants aged between 40 and 69 were selected from GP lists and invited to attend a center, where blood, urine and saliva samples were taken, physical measurements were made (eg, blood pressure, anthropometric measurements), and extensive health and lifestyle questionnaires were completed.

DNA was extracted from buffy coat at UK Biocenter (Stockport, UK) using a Promega Maxwell® 16 Blood DNA Purification Kit (AS1010). Samples with sufficient DNA concentration and purity (as measured by 260/280 ratio) were aliquoted and 50 μL were shipped for genotyping at Affymetrix (Santa Clara, Ca, USA). A bespoke sample selection algorithm was used to ensure that the samples on each plate were from participants from a range of recruitment centers.

#### The UK BiLEVE Study

The UK Biobank Lung Exome Variant Evaluation (UK BiLEVE) study involves a subset of 50,008 participants from UK Biobank, selected to investigate the genetic determinants of smoking behavior, lung function and chronic obstructive pulmonary disorder (COPD) (Wain et al., 2015). The UK BiLEVE participants included equal numbers of males and females selected from those who self-reported being of white European ancestry, had sufficient spirometric measurements to determine lung function measures, were either never smokers or ‘heavy smokers’ (mean 35 pack years), and had either poor lung function, average lung function or high lung function. As the UK BiLEVE participants are a subset of the UK Biobank study, DNA extraction, aliquoting and shipment procedures were as described above.

#### The INTERVAL Study

The INTERVAL study is a prospective cohort study of approximately 50,000 participants nested within a pragmatic randomized trial of blood donors (Moore et al., 2014). Between 2012 and 2014, blood donors 18 years and older were consented and recruited from 25 NHSBT (National Health Service Blood and Transplant) static donor centers across England. Participants are predominantly healthy individuals since people with major disease (myocardial infarction, stroke, cancer etc) are ineligible for donation, as are those who report being unwell or having had recent illness or infection.

Participants completed online questionnaires containing basic lifestyle and health-related information, including self-reported height and weight, ethnicity, current smoking status, alcohol consumption, doctor-diagnosed anemia, use of medications (hormone replacement therapy, iron supplements) and menopausal status.

DNA was extracted from buffy coat at LGC Genomics (UK) using a Kleargene method and samples of sufficient concentration and purity were aliquoted for shipment to Affymetrix for genotyping. A modified version of the sample selection algorithm used for the UK Biobank study was implemented to ensure that samples on each plate came from participants with a mix of recruitment center, recruitment date, regional hub and gender.

The INTERVAL study was approved by the Cambridge (East) Research Ethics Committee and UK Biobank was approved by the North West Multi-center Research Ethics Committee (MREC). Informed consent was obtained from all participants.

### Method Details

#### The UK Biobank and UK BiLEVE Affymetrix Axiom Genotyping Arrays

The UK Biobank Affymetrix Axiom array is a customized genotyping array comprising 845,485 probesets assaying 820,967 single nucleotide variants (SNVs) and short insertions/deletions (indels; http://www.ukbiobank.ac.uk/scientists-3/uk-biobank-axiom-array/). The array includes an “exome” component, designed to capture variants likely to have transcriptional consequences (nonsynonymous, splice altering, truncating), and a “genome-wide association study (GWAS) scaffold” selected to ensure good quality genome-wide imputation of variants that are common (minor allele frequency [MAF] > 5%) or low-frequency (MAF = 1%–5%) in European populations. The exome component, which includes approximately 130,000 (predominantly rare) variants, was designed using data from three large exome sequencing projects: the NHLBI Exome Sequencing Project (Tennessen et al., 2012), the Exome Aggregation Consortium (ExAC) (Minikel et al., 2016) and the UK10K project (UK10K Consortium et al., 2015). Additional rare variants were included in cardiac disease and cancer predisposition genes, as well as other variants from the Human Gene Mutation Database (HGMD) (Stenson et al., 2014).

The genome-wide imputation scaffold was designed by selecting tagging variants from Affymetrix databases using a custom algorithm. In addition to 246,000 variants from the 1000 Genomes CEU population designed to tag common variants in European populations, an additional 103,000 variants from additional European 1000 Genomes populations were added to boost imputation of common variants, as well as a further 280,000 variants to boost imputation in the UK population in the 1%–5% MAF range. Mean *r*^2^ between observed and imputed genotypes for common variants was estimated to be 0.92, while for low-frequency variants it was 0.79 (http://biobank.ctsu.ox.ac.uk/crystal/refer.cgi?id=155580), suggesting that the array was able to impute lower frequency variants with greater accuracy than previous GWAS arrays typically could.

The remaining content on the array includes markers of specific relevance, including markers related to diseases and traits (Alzheimer’s, autoimmune and inflammatory, blood phenotypes, cancers, cardiometabolic, neurological disease), dense coverage of selected genomic regions (HLA, ApoE, KIR, Y chromosome, mitochondria, copy number variants relevant to certain conditions) and other categories (variants related to gene expression, fingerprint markers, tags for Neanderthal ancestry, and pharmacogenetic markers). Of particular relevance to this study, the array included 2,545 variants related to blood and iron phenotypes, including red cell blood groups, regulation of hematopoiesis (red blood cells, platelets, white blood cells) and regulation of blood homeostasis identified from candidate gene studies, GWAS and review of the literature.

The UK BiLEVE Affymetrix Axiom genotyping array preceded the UK Biobank array and was designed similarly (overlapping content > 95%). Due to the focus of the UK BiLEVE study, their array contained content designed to genotype or tag variants known or suspected to be related to lung function or disease, COPD, asthma or smoking behavior. In total, the array had 833,090 probesets assaying 807,411 variants. The 50,008 participants in the UK BiLEVE study were genotyped on the UK BiLEVE array, while the remaining UK Biobank participants and the INTERVAL participants were genotyped on the UK Biobank array.

#### Genotyping

For all three studies, aliquots were shipped to Affymetrix in 96-well barcoded plates with two empty wells for Affymetrix controls. Samples were quantified using a PicoGreen-based method to identify plates with high numbers of low concentration samples, which could be replaced prior to genotyping. Genotyping was performed on the Affymetrix GeneTitan® Multi-Channel (MC) Instrument according to the Affymetrix Axiom 2.0 Assay Automated Workflow. Genotypes were then called in batches of approximately 50 plates (∼4800 samples) using the Affymetrix Power Tools software to implement the Axiom GT1 algorithm. For the UK Biobank and UK BiLEVE studies, rare variants (i.e., those with fewer than six minor alleles in a genotyping batch) were recalled using variant-specific priors to improve performance.

#### Quality Control (QC) of Genotype Data

For all studies, Affymetrix implemented standard QC procedures during the genotype calling pipeline, excluding samples with poor signal intensity (dish QC < 0.82) and samples with low call rate (< 97%) based on ∼20,000 high quality probesets. Variants were excluded if they had low call rate (< 95%), had more than three clusters (indicative of off-target measurement), had cluster statistics (Fisher’s linear discriminant, heterozygous cluster strength, homozygote ratio offset) indicative of poor quality genotyping or were complicated multi-allelic variants that couldn’t easily be called.

##### QC of UK BiLEVE Genotype Data

As UK BiLEVE participants were genotyped prior to the other UK Biobank participants on a slightly different array, QC of UK BiLEVE genotyping data was carried out separately by UK BiLEVE investigators (Wain et al., 2015). Briefly, a total of 50,561 samples were genotyped in eleven batches. Samples were excluded if they were sex mismatches, unresolvable duplicates (> 98% of alleles identical by descent [IBD]), heterozygosity outliers (greater than three standard deviations [SD] from the mean), ethnic outliers (greater than ten SD from the mean on any of the first ten principal components (PCs) generated including all HapMap3 panels (International HapMap 3 Consortium et al., 2010), or had withdrawn consent. Intentional duplicate pairs and related individuals (IBD > 20%) were resolved, excluding individuals with the highest number of pairwise relationships then the lowest call rate. After these steps, 48,943 participants remained for analysis.

For variants with multiple probesets, only the probeset with the highest call rate was retained. Variants were additionally excluded from a batch if they showed within-batch plate effects (p value < 1x10^−6^) and variants that failed in more than two of the eleven batches were dropped from the dataset. A total of 782,260 variants remained after QC.

##### QC of UK Biobank Genotype Data

At the time of submission of this paper, genotyping data were available on the first ∼150,000 participants from the UK Biobank study, including the ∼50,000 participants selected for the UK BiLEVE study. QC of UK Biobank genotyping data from these participants, carried out by UK Biobank investigators, has been described in detail elsewhere (http://biobank.ctsu.ox.ac.uk/crystal/refer.cgi?id=155580). In total, 153,293 samples were genotyped across 33 batches. Samples with high missingness or high heterozygosity (accounting for ethnicity) were excluded based on visual inspection of ancestry-specific plots, as were samples from participants who had withdrawn. A further eight samples who had low heterozygosity that couldn’t be explained by long runs of homozygosity were also excluded. For variants with multiple probesets, the probeset defined by Affymetrix as “best” was retained. Variants showing batch effects (either within the UK Biobank batches or between UK Biobank and UK BiLEVE batches), within-batch plate effects, or within-batch deviations from Hardy-Weinberg equilibrium (HWE) in European ancestry samples defined by principal component analysis (PCA), all at p value < 1x10^−12^, were filtered from the batches in which they failed. In total, after these exclusions, data were available for 151,733 participants on 806,466 variants that passed in at least one batch.

##### Additional QC of UK Biobank and UK BiLEVE Genotype Data

In addition to the QC steps applied by UK Biobank and UK BiLEVE investigators, we implemented sample filtering on the combined dataset. We excluded samples with more than 3% missingness, samples with missing phenotypic sex, and samples with sex mismatches or dubious sex estimation from the genotyped data. To restrict analyses to participants of European continental ancestry, we defined a ‘genetic distance’ *d (i)* between individual *i* and a hypothetical median “white British” participant using variance weighted PC scores,

$$d\left(i\right)=\sqrt{\sum_{m=1}^{15}E_{m}{\left(P_{im}-C_{m}\right)}^{2}}$$

Where:

*m* is an index for each of the 15 PCs provided by UK Biobank,

*E*_*m*_ represents the eigenvalue corresponding to PC *m (*i.e., the genetic variance explained by PC *m*)

*P*_*im*_ represents the score of individual *i* on PC *m*

*C*_*m*_ represents the median score on PC *m* of participants with self-reported White ancestry (defined as “British,” ”Irish,” “White,” or “Any other White background”)

We used a threshold of genetic distance > 50 to identify non-Europeans, which resulted in the exclusion of 7,848 non-European samples.

To implement further QC steps (heterozygosity analysis, PCA and identification of duplicate samples), a robust set of variants were derived using the same methods as UK Biobank, i.e., selecting autosomal variants on both arrays that had passed variant QC in all 33 batches, had MAF ≥ 2.5% and missingness ≤ 1.5%, were not indels, were not C/G or A/T variants, and were not within 23 regions of known long-range linkage disequilibrium (LD). These variants were then LD-pruned (*r*^2^ < 0.1) to obtain an uncorrelated set of variants. The first fifty PCs were estimated using flashpca (Abraham and Inouye, 2014) and the heterozygosity analysis, which was carried out in parallel with the ethnic outlier identification using PLINK v1.9 (Chang et al., 2015), identified 3,030 samples that had autosomal heterozygosity greater than three SD from the mean, 2,667 of which were also ethnic outliers. To identify duplicate samples, we performed identity-by-descent (IBD) analysis using the PLINK Method-of-Moments approach (http://pngu.mgh.harvard.edu/∼purcell/plink/ibdibs.shtml), which identified 19 pairs of duplicate/monozygotic twins (pi_hat ≥ 0.9). All 38 samples were excluded from the analysis dataset.

##### Quality control (QC) of INTERVAL Genotype Data

In total, 48,813 INTERVAL samples were genotyped in ten batches. Following standard Affymetrix QC exclusions, within-batch sample and variant QC was performed. Non-best probesets were excluded to leave a single probeset per variant. As visual inspection of cluster plots had identified that some variants, particularly rare variants, had minor allele homozygotes incorrectly called due to the presence of an extreme intensity outlier, we failed variants from a batch if:

• the variant had fewer than ten called minor allele homozygotes;
• the cluster plot contained at least one sample with an intensity at least twice as far from the origin as the next most extreme sample;
• the outlying sample (s) had an extreme polar angle (< 15° or > 75°) in the direction of the minor allele.

Prior to further QC of variants within each batch, we excluded duplicate samples and samples that were clearly not of European ancestry using a set of high-quality autosomal variants, defined as those with:

• MAF > 0.05
• HWE p value > 1x10^−6^
• *r*^2^ ≤ 0.2 between pairs of variants.

Duplicate samples were defined as those with $\hat{\pi }$ ≥ 0.9 using the PLINK Method-of-Moments IBD approach and non-Europeans were defined as those with scores on PC1 or PC2 < 0 following a PCA including INTERVAL samples with 1000 Genomes major ancestry populations (1000 Genomes Project Consortium et al., 2015).

Variants were then excluded from a batch if they strongly deviated from HWE (p value < 5x10^−6^), following a Fisher’s exact test for low-frequency and rare variants (defined as those with a maximum MAF < 0.05 across all ten batches) or a $\chi^{2}$ test for common variants. Similarly, variants were excluded from a batch if they had a within-batch call rate < 0.97. Finally, variants were dropped from all batches if they failed in at least four of the batches due to deviation from HWE, low call rate or Affymetrix variant exclusion criteria.

After merging passing samples and variants across the ten batches, we estimated the level of sample contamination using the method described by Jun et al. (2012), which examines the relationship between allele frequency and probeset intensity. We excluded samples with more than 10% contamination, as well as those who had both 3%–10% contamination and ten or more first- or second-degree relatives (defined as pi_hat ≥ 0.1875). Heterozygosity outliers (heterozygosity more than three standard deviations away from the mean), samples with missing phenotypic sex and sex mismatches were then also removed, as were variants with a MAF range greater than 0.05 across all batches, variants that were monomorphic in one or more batches but had MAF > 0.01 in another batch, and variants that had different minor alleles between batches (only for variants with maximum MAF < 0.475 across batches).

For IBD analysis and PCA, another set of ∼100,000 high quality variants was selected using the same criteria described above for the UK Biobank QC (Figure S3). The global IBD analysis (performed using PLINK Method-of-Moments approach) revealed 69 pairs of across-batch duplicates (or monozygotic twins), who were removed from the dataset. A between-study IBD analysis, including the INTERVAL, UK Biobank and UK BiLEVE studies revealed a further 1100 participants who were in both the INTERVAL and combined UK Biobank-UK BiLEVE datasets, so these participants were excluded from the INTERVAL dataset to avoid overlap. The PCA, performed using flashpca without including 1000 Genomes samples, identified a further twelve outliers who leveraged lower PCs (PC 6, 8 and 9) according to a visual check and were therefore excluded from the dataset. The PCA was then re-run to obtain final PCs for use as covariates in analysis models. 43,059 participants remained in the final dataset.

#### Variant Imputation

##### UK Biobank and UK BiLEVE

The pre-imputation variant QC, phasing and imputation conducted on the combined UK Biobank and UK BiLEVE dataset has been described in detail (http://biobank.ctsu.ox.ac.uk/crystal/refer.cgi?id=157020). Briefly, sample and variant QC was performed as described above, then variants were additionally removed if they:

• were only on the UK BiLEVE array and had failed in more than one (of eleven) UK BiLEVE batches;
• were only on the UK Biobank array and had failed in more than two (of 22) UK Biobank batches;
• were on both arrays and had failed in three or more of the 33 total batches.

Multiallelic variants and variants with MAF < 0.01 were then removed, as were non-autosomal variants. The UK Biobank and UK BiLEVE study samples were then jointly phased and imputed using a combined 1000 Genomes Phase 3-UK10K panel. Phasing was conducted using SHAPEIT3 (O’Connell et al., 2016), a modified version of SHAPEIT2 (Delaneau et al., 2013), in chunks of 5,000 variants with an overlap of 250 variants between chunks. Imputation was performed using IMPUTE3, a modified version of the IMPUTE2 software (Howie et al., 2011), in chunks of 2Mb with a 250kb buffer region. Post-imputation, variants with MAF < 0.00001 (1 in 100,000) were filtered from the dataset using QCTOOL (http://www.well.ox.ac.uk/∼gav/qctool/), leaving 72,355,667 variants for analysis in the dataset.

##### INTERVAL

Prior to imputation, additional variant QC steps were performed to establish a high quality imputation scaffold. We imposed a global HWE filter of p value < 5x10^−6^, a call rate filter of 99% over the batches that a variant was not failed in, and a global call rate filter of 75% (effectively ensuring a variant passed in at least eight of the ten batches). Finally we removed all monomorphic variants.

Non-autosomal and multi-allelic variants were removed as part of the QC process and the dataset was then phased using SHAPEIT3, with the same criteria used for UK Biobank (chunks of 5,000 variants with an overlap of 250 variants between chunks) and subsequently imputed using the same combined 1000 Genomes Phase 3-UK10K imputation panel described above. Imputation was performed on the Sanger Imputation Server (https://imputation.sanger.ac.uk), which uses the PBWT imputation algorithm (Durbin, 2014), and analyses whole chromosomes. No imputation quality or variant frequency filters were applied, resulting in 87,696,910 imputed variants in the dataset.

Using whole-exome sequencing (WES) data for 3,976 INTERVAL study participants who were also in our post-QC imputation dataset, we were able to assess imputation accuracy. We adapted two metrics (Linderman et al., 2014) to compare genotype data to sequencing data for these purposes. The first was non-reference concordance, which considers all heterozygotes and minor allele homozygotes in the WES dataset and calculates the proportion seen in the imputed dataset. The second was precision, which considers all the heterozygous and minor allele homozygotes in the imputed dataset, and calculates what proportion of these calls was correct according to the WES dataset. For 146 missense, loss-of-function or rare high-impact (beta > 0.5SD) variants that passed QC in the WES dataset, we observed a median non-reference concordance of 98.6%, 98.8% and 93.9% in common (MAF > 0.05), low-frequency (MAF > 0.01 and MAF ≤ 0.05) and rare variants (MAF < 0.01) respectively and median precision of 99.5%, 99.3% and 98.5% in common, low-frequency and rare variants respectively.

#### Phenotype Measurement, QC, and Processing

##### Variability of Hematological Indices

We studied 36 hematological traits in individuals of European ancestry selected from the UK Biobank and INTERVAL studies (Figure 1; Table S1). The traits comprise the main hematological indices of the seven types of cells reported in a standard clinical full blood count (FBC) analysis and additional variables derived from them (Table S2), measuring properties of mature and immature red blood cells (twelve indices), platelets (four indices) and myeloid and lymphoid white blood cells (twenty indices). The indices include cell counts per unit volume of blood (e.g., the counts of the six types of myeloid cells and lymphocytes), ratios of cell counts (e.g., count of neutrophils as the percentage of myeloid white cells), mean volume of platelets and red cells (MPV and MCV, respectively), proportions of blood volume occupied by cells (e.g., hematocrit) and measurements of the concentration and mass distribution of cellular hemoglobin (e.g., mean corpuscular hemoglobin [MCH]).

Exploiting extensive metadata on the blood cell index measurements and anthropometric covariates, we performed thorough quality control to identify and remove sources of technical and non-genetic biological variation, increasing our power to detect genetic associations. Technical covariables such as the time between venipuncture and FBC analysis, FBC instrument drift and calibration events and episodes of malfunction, explained up to 16% of the variance of each index (Figure S1). Further, non-technical sources of covariation such as age, sex and menopause status were shown to affect blood cell indices strongly, accounting for up to 40% of variance in the residuals after adjustment for technical factors (Figure S2). We made flexible adjustments for age within sex and menopause categories using semi-parametric regression. Additionally, using clinical knowledge, we selected a subset of measured covariates to screen for association with indices in the UK Biobank dataset. Body mass index and variables measuring smoking habits and alcohol consumption covariates explained at least 0.5% of variance in one or more of red cell, platelet or white cell indices after adjustment for age and sex, and were thus included in our adjustments.

##### Measurement of Blood Cell Indices

Full blood counts were measured in UK Biobank and INTERVAL study participants using clinical hematology analyzers at the centralized processing laboratory of UK Biocenter (Stockport, UK). Research blood samples for the baseline assays of UK Biobank volunteers were collected into 4ml EDTA vacutainers by vacuum draw at the UK Biobank assessment centers and were then stored at 4 degrees centigrade. The samples were transported overnight to UK Biocenter in temperature-controlled shipping boxes.

For the INTERVAL baseline assays, research blood samples were taken from each participant through the satellite pouch of a blood collection unit, with the venipuncture performed as part of a routine NHSBT whole blood donation (Moore et al., 2014). The samples for FBC analysis were collected in 3ml EDTA tubes and were transported to NHSBT holding sites (‘hubs’) at Manchester, Colindale (London) and Bristol, from where they were taken overnight by courier to UK Biocenter. The INTERVAL blood samples were kept predominantly at ambient temperature from the time of collection to the time of measurement.

At UK Biocenter, the UK Biobank whole blood samples were processed using four Beckman Coulter LH700 Series instruments while the INTERVAL samples were processed using two Sysmex XN-1000 instruments. The two models of analyzer both measure full blood counts by a combination of fluorescence and impedance flow cytometry. However, there are some differences in the cytometric methods used by the instruments to distinguish and count the different types of blood cell. The different analysis techniques require different manufacturer-supplied reagents to treat, lyse and fluoresce the cells, which can result in differences in the measurement response.

##### Technical Sample Exclusions

As a blood sample ages, the accuracy of a full blood count (as a measurement of the properties of peripheral blood at the time of venipuncture) deteriorates. The exact consequences of sample aging depend on the measurement techniques used by the instrument. For example, blood cell membranes become more elastic as a sample ages. Consequently, if the analyzer uses a hypotonic solution, cells in an older sample tend to swell more at the point of measurement than cells in a younger sample. This excess swelling leads to bias in the measurement of traits determined as a function of plateletcrit (PCT) or hematocrit (HCT) (Ulset et al., 2014). It may also become more difficult to differentiate between the types of white cell as a sample ages and very old samples are likely to suffer from hemolysis (Sowemimo-Coker, 2002).

Greater than 99% of UK Biobank baseline FBCs and 98% of INTERVAL baseline FBCs were measured fewer than 48 hr after venipuncture. Respectively 72% and 75% of the FBCs were measured fewer than 24 hr after venipuncture. Although clinical laboratories do not usually issue FBC reports measured on samples aged for more than about 12 hr, FBCs from blood samples below clinical standard may still add useful information to genetic association analysis. However there is a tradeoff; the inclusion of very noisy samples may reduce power if the marginal increase in sample size is insufficient to compensate for the reduction in signal to noise ratio. Consequently, we excluded participants from the association analysis if they had FBCs measured more than 36 hr after venipuncture. This removed 11,490 participants from the UK Biobank phenotype dataset, 3,365 of whom had been genotyped, and 1,490 participants from the INTERVAL phenotype dataset.

The Coulter analyzers distinguish platelets from red cells by impedance (Table S1), a proxy for cell volume. Consequently small red cells can sometimes be confused with large platelets. Sysmex analyzers also use impedance to measure platelet volume, but they measure platelet count by both fluorescence flow cytometry and impedance and routinely report the former measurement. Sysmex instruments flag measurements of mean platelet volume (MPV) greater than 13 as unreliable on the grounds that the large volume measurements suggest contamination of the platelet impedance channel by red cells. We excluded such data points from the INTERVAL analysis. In order to similarly protect against contamination of platelet variables by red cells in the UK Biobank dataset, we removed all platelet trait data from FBCs with a technically adjusted MPV measurement larger than the 96th percentile.

##### Blood Cell Index Data Adjustments

In order to optimize the power to detect allelic associations, we adjusted the baseline blood cell index values from the INTERVAL and UK Biobank datasets to remove variance explained by technical, environmental and sex effects. We adjusted the data from the INTERVAL and UK Biobank studies independently because of differences between the study populations, the differences between the sample collection protocols and the use of different models of hematology analyzer. At the time we carried out the analyses described in the present publication, the UK Biobank study investigators had released genetic data for approximately one third of the cohort, which included the UK BiLEVE participants as a subset. However, we chose to adjust the phenotypes from the entire UK Biobank baseline blood indices dataset (n = 476,675) in order to estimate covariate effects as precisely as possible. Our phenotypic adjustments are more extensive than has hitherto been usual for genome-wide association studies. Covariate adjustment absorbs variance (i.e., “uses up degrees of freedom”) and we do not model this directly in the association analyses. However, we do use Genomic Control (see below), which corrects the test statistics for this modelling omission.

We made the adjustments differently by blood cell index and by analyzer model according to whether the index was *measured* or *derived*. For each analyzer model the *measured indices* are a minimal subset of indices from which the full set of indices can be deterministically calculated (Table S1). These subsets were chosen, from all possible minimal subsets, to correspond as closely as possible to direct independent measurements made by the analyzers. The *derived indices* are the indices complementary to the measured indices and which can therefore be calculated from them.

We made the blood cell index adjustments in two stages. In the first stage we removed technical outliers and independently adjusted each measured index for technical and seasonal covariates. We then recomputed the derived indices from the measured indices. In the second stage we adjusted both measured and derived indices for non-seasonal environmental covariates and for sex.

FBC indices divide into variables that have a population distribution with positive support (counts and concentrations), and variables that have a population distribution with support in [0,1] or [0%,100%] (cell count ratios and volume proportions). We adjusted the positively supported indices on the log-scale and the proportion-supported indices on the logit scale. We call these scales the *adjustment-scales* for the indices. To adjust platelet distribution width and red cell distribution width, we computed the standard deviations of the platelet volume and red-cell volume distributions, adjusted these on the log-scale and then recomputed the distribution widths as coefficients of variation.

##### Technical and Seasonal Variance

Clinical FBCs, like all assays, are subject to measurement error. Moderate technical variation in FBCs is rarely a concern for clinicians who use FBC reports to diagnose or exclude blood pathologies that cause a large deviation in a measured blood cell parameter from its typical population value. However, the power of quantitative trait association analysis depends monotonically on the proportion of variance explained by the associated allele. It is therefore important to remove as much technical variation from the measured trait values as possible.

By visual inspection of within-instrument window-averaged time series for each blood cell index (e.g., plots of mean index value by day of study, by week of study within machine, by time of day within machine) we identified for some or all of the measured indices for both studies the following sources of technical variance (Figures S1 and S2):

• differences in the average index value by instrument
• short time periods during which the day-averaged value of the instrument reading deviated dramatically from the global average value for the instrument over the duration of the study, probably due to temporary aberrant behavior of the instrument
• continuous long term drift in the average index value reported by the instrument over time
• time-discontinuities in average index values probably due to calibration events
• variation in the average index value by time of year
• variation in the average index value by age of sample i.e., time between venipuncture and measurement
• variation in the average index value by the time of day of measurement.

For each blood cell index, we used the central part of the data (the data differing from the median by less than 3.5 median absolute deviations on the adjustment scale) to estimate the effect on the mean of the (adjustment scale transformed) index of within machine time-dependent drifts, delay time between venipuncture and measurement, day of the week and time of year. We restricted the model fit to the central part of the data in order to minimize influence from outlying data points. After fitting the regression model we computed model residuals for the full dataset and used these residuals to compute index values adjusted for technical effects.

Specifically, we used the R package *mgcv* (https://cran.r-project.org/package=mgcv) (R Core Team, 2014, Wood, 2011) to fit a generalized additive model (GAM) with the following regression equation:

$$E\left(a\left(y_{i}\right)\right)=s\left[t\left(i\right)⊗m\left(i\right)\right]+s\left[\left(t_{\text{day}}\left(i\right),t_{\text{ven}}\left(i\right)\right)⊗m\left(i\right)\right]+\sum_{\underset{\left\{\text{mon,}\ldots \text{,sun}\right\}}{w\in }}\left[1_{w\left(i\right)=w}\right]+c\left[t_{\text{year}}\left(i\right)\right]+\sum_{m}\left[1_{m\left(i\right)=m}\right]$$

Here:

• *a* denotes a function transforming the measured index data *y* to the adjustment scale.
• *m* (*i*) denotes the instrument used to acquire measurement *i.*
• *w* (*i*) denotes the day of the week on which measurement *y*_*i*_ was acquired.
• *t* (*i*) denotes the time difference between the time of measurement of observation *i* and midnight (am) on the first day of the study.
• *t*_year_ (*i*) represents the difference between the time of measurement of observation *i* and midnight (am) on the 1^st^ of January on the year in which observation *i* was measured.
• *t*_day_ (*i*) represents the difference between the time of measurement of observation *i* and midnight (am) on the day of measurement.
• *t*_ven_ (*i*) represents the difference between midnight *(*am) on the day observation *i* was measured and the time of venipuncture.
• Each term in square brackets represents a contribution to the linear predictor.
• s[] indicates a smoothing term. For the univariate terms we smooth using P-splines, while for bivariate smooths we smooth using thin plate splines.
• c[] indicates a cyclic smoothing term, used here to model seasonal variation on a circle representing time of year.
• We use the symbol ⊗ to indicate the presence of an interaction between the smooth and the categorical variable to its right (in both cases here, the instrument id *m* (*i*)).

The first term in the regression equation models long term drift effects, for which we fit a smooth with 50 knots, allowing a different drift model within each machine. The second term (bivariate in *t*_day_ (*i*) and *t*_ve_ (*i*) and for which we fit a smooth with 30 knots) jointly absorbs variation due to mean drift within machine over the course of a day and mean drift caused by the time delay between venipuncture and measurement. The cyclic term (30 knots) models seasonal effects for which we force consistency across the instruments. The dummy variable terms model mean differences by day of the week and machine.

After making the adjustment for drift we sought to remove data-points due to periods of aberrant operation. After transforming the index data to the adjustment scale, we computed a standardized score *z*_*d,m*_ to measure the deviation of the day (*d*) and instrument (*m*) specific average trait values from the global mean value:

$$z_{d,m}=\sqrt{\left|\#\left\{a\left(y_{i}\right):m\left(i\right)=m,d\left(i\right)=d\right\}\right|}\times \frac{\left|\text{mean}\left\{a\left(y_{i}\right):m\left(i\right)=m,d\left(i\right)=d\right\}-\text{median}\left\{a\left(y_{i}\right)\right\}\right|}{\text{median}\left\{\left|a\left(y_{i}\right)-\text{median}\left\{a\left(y_{i}\right)\right\}\right|\right\}}$$

Here:

• *a* (*y*_*i*_) represents the trait data for observation *i* on the adjustment-scale, after correction for drift using the GAM.
• *d* (*i*) denotes the day on which the index measurement for *i* was acquired.
• Measurements acquired on day-instrument pairs with fewer than 10 data-points or for which *z*_*d,m*_ > 8 were excluded from further analysis.

After making these exclusions we refitted the GAM for drift described above to obtain measured index values that are adjusted for drift effects without the influence of data from aberrant days. We then recomputed the derived indices from the measured indices. For some indices, the power gained from the adjustments for technical effects alone is equivalent to thousands of additional samples (Figure S1).

##### Exclusions Based on Phenotypes and Covariates

We sought to exclude individuals with blood cancers or major blood disorders from the UK Biobank study on the grounds that, if included, their noisy blood counts may reduce the power to detect genetic associations. Using data from the baseline health assessment self-report, the linked cancer registry and linked hospital inpatient record summaries, we identified and removed individuals suffering from blood cancers or other blood disorders. Specifically we excluded participants who had a self-report or medical history containing a record of myelofibrosis, lymphoma, leukemia, malignant lymphoma, multiple myeloma, multiple myelofibrosis or myelodysplasia, chronic lymphocytic leukemia, chronic myeloid leukemia, acute myeloid leukemia, polycythemia vera, polycythemia, a myeloproliferative disorder, essential thrombocytosis, a hematological cancer histology report, an unspecified lymphatic or general hematological neoplasm, a myelodysplastic syndrome, or an unspecified heme malignancy, monoclonal gammopathy, an unspecified hereditary hematological disorder, hemochromatosis, thalassemia, hemophilia, sickle cell anemia, neutropenia, lymphopenia or pancytopenia. In aggregate this excluded 5,045 participants from the UK Biobank phenotype dataset, of whom 1,611 had measured genotypes.

Since we had no access to detailed health record data on the INTERVAL participants, we did not make any similar exclusion for INTERVAL. However, participants in the INTERVAL study are generally healthier than those in UK Biobank and are active whole blood donors, therefore the incidence of blood disorders is likely to be substantially lower. Hematologists screened the baseline full blood counts of INTERVAL participants and very few probable cases of leukemia were identified.

##### Non-seasonal Environmental and Variance Explained by Sex Differences

We adjusted all indices for environmental and sex differences using a GAM, again solely using the central part of the data (the data after adjustment for technical effects, differing from the median by less than 3.5 median absolute deviations on the adjustment scale) to fit the model. The measured environmental covariates differ between the INTERVAL and UK Biobank studies and consequently the models we fitted differed slightly.

For the INTERVAL study dataset we fit a model with the following terms:

• A univariate smooth (30 knots) for age at venipuncture, with an interaction with a categorical variable describing menopausal status with the following levels: male, female-premenopausal, female-postmenopausal, female-had-hysterectomy, no-answer, unsure
• A bivariate smooth (30 knots) for log-height and log-weight (which implicitly adjusts for body-mass index [BMI]), with the same categorical interaction variable as for age
• A univariate smooth for pack-years of smoking
• A categorical variable describing current smoking habits with levels: never, special-occasions, rarely, occasional, most-days, every-day, no-answer
• A categorical variable describing alcohol drinking status with levels: never, previous, current, no-answer
• A categorical variable describing current alcohol drinking habits with levels: never, special-occasions, 1-3-times-monthly, 1-2-times-weekly, 3-5 times weekly, most-days, no-answer.

For the UK Biobank study dataset we fit a model with the following terms:

• A univariate smooth (30 knots) for age at venipuncture, with an interaction with a categorical variable describing menopausal status with the following levels: male, female-premenopausal, female-postmenopausal, female-had-hysterectomy, unsure
• A univariate smooth (30 knots) for number of days since last period (within women only)
• A bivariate smooth (30 knots) for log-height and log-weight (which implicitly adjusts for BMI), with the same categorical interaction variable as for age
• A univariate smooth (30 knots) for quantity of alcohol consumed the day prior to recruitment
• A univariate smooth for pack-years of smoking
• A categorical variable describing current smoking habits with levels: never, special-occasions, rarely, occasional, most-days, every-day, no-answer
• A categorical variable describing alcohol drinking status with levels: never, previous, current, no-answer
• A categorical variable describing current alcohol drinking habits with levels: never, special-occasions, 1-3-times-monthly, 1-2-times-weekly, 3-5 times weekly, most-days, no-answer.

For both datasets, where data-points were missing for a covariate, we imputed them by the mean covariate value and included a dummy variable to allow the mean of the index value for individuals with missing data to differ from the mean index value for individuals with non-missing data.

##### Removal of Outliers and Normalization

We removed observations by index for which there was a large difference between the raw measured index value and the adjusted index value. Specifically, we removed a data point if the difference, on the adjustment scale, between the original raw measured data and the adjusted data was more than 3.5 median absolute SD from the median of the distribution of such differences for the relevant index.

We removed outliers from the phenotype data. We first considered outliers in each marginal univariate distribution. For each index on the adjustment scale, we removed all data-points lying more than 4.5 median absolute deviations from the median index value on the adjustment-scale. We then grouped the indices as follows:

• MPV, PLT#, PDW, PCT (platelet traits)
• HGB, RBC#, MRV, MCV, MCH, MCHC, RDW, RDW, RET#, HLR, HCT, RET%, HLR%, IRF (mature and immature red cell traits)
• RET, HLR, RET%, HLR%, IRF (immature red cell traits)
• WBC#, NEUT#, MONO#, BASO#, EO#, LYMPH#, MYELOID, GRAN#, (EO+BASO)#, (NEUT+EO)#, (BASO+NEUT)#, NEUT%, EO%, MONO%, LYMPH%, BASO%, GRAN%MYELOID, EO%GRAN, NEUT%GRAN, BASO%GRAN (white cell traits)
• NEUT#, BASO#, EO#, GRAN#, (EO+BASO)#, (NEUT+EO)#, (BASO+NEUT)#, EO%GRAN, NEUT%GRAN, BASO%GRAN (granulocyte traits)
• NEUT#, MONO#, BASO#, EO#, MYELOID, GRAN#, (EO+BASO)#, (NEUT+EO)#, (BASO+NEUT)#, GRAN%MYELOID, EO%GRAN, NEUT%GRAN, BASO%GRAN (myeloid white cell traits)
• all traits

After standardizing the variables on the adjustment scale, we performed a principal component analysis for each group and computed the sum of squares of the leading *d* PC-scores where *d* is the number of independent measurements required to compute the variables in each group. We compared the sum of squares to a *χ*^*2*^_**d**_ distribution and removed outliers falling into the upper 10^−7^ tail probability.

Finally, within each study we quantile-inverse-normal transformed the trait data within each level of a categorical variable formed by crossing a categorical variable indexing the hematology analyzer with a categorical variable with the levels male, female-premenopausal, female-postmenopausal, female-had-hysterectomy, no-answer, unsure.

The final number of participants passing phenotype and genotype QC from each of the studies is shown in Table S2, along with summary statistics for each blood cell index.

### Quantification and Statistical Analysis

#### Association Tests, Meta-Analyses, and Identification of Distinct Associations

##### Univariable GWAS

Genetic and phenotypic QC retained 173,480 participants (87,265 in the UK Biobank study dataset, 45,694 in the UK BiLEVE study dataset and 40,521 in the INTERVAL study dataset). We performed a univariable GWAS for each of the 36 blood cell indices that had phenotype data measured or derived in all three studies. Specifically, we computed the association statistics (i.e an estimate of the regression coefficient and the corresponding standard error) for a mixed linear regression of phenotype on the probabilistic imputed allele dose (i.e., an additive model) separately for each of the three datasets using BOLT-LMM v2.2 (Loh et al., 2015). The linear mixed model accounts for the genetic component of phenotypic correlation generated by relatedness. In order to maximize protection against confounding by large scale relatedness, we included a dummy variable for each recruitment center and the first ten PCs of the study specific kinship matrices as covariates in each regression model.

##### Meta-Analyses and Significance Threshold

Having performed univariable GWAS within each study, we then combined the results across the three studies using meta-analysis. For inclusion in the meta-analysis, a variant had to have a study-specific MAF > 0.01%, an imputation dataset-specific information score greater than 0.4, and non-missing effect size estimates and standard errors in all three datasets. 29.5 million variants were retained. We performed an inverse variance weighted meta-analysis using METAL (Willer et al., 2010). To guard against confounding by unmodeled relatedness, we performed double genomic control to adjust the pre and post meta-analyses standard errors for variance inflation, with respect to a genome-wide null assumption. The inflation factors were estimated as ratios of the median of the observed $\chi_{1}^{2}$ test statistics to the median of the $\chi_{1}^{2}$ distribution. Finally, using the meta-analyses summary statistics, we performed a Wald test for each blood cell-index variant pair against the null hypothesis of no additive allelic association. We used the significance level α = 8.31x10^−9^, a threshold recently estimated for genome-wide analyses of common, low frequency and rare variants (UK10K Consortium et al., 2015, Xu et al., 2014).

##### Heterogeneity Filtering

Substantial statistical evidence for heterogeneity in effect sizes between the studies of a meta-analysis for a genome-wide significant variant is often taken to suggest a false-positive association. However, effect size heterogeneity in GWAS can be generated by:

• population-genotype interactions (i.e., true allelic effect size differences between studies),
• variation in LD between study populations,
• study specific quantile-inverse-normal transformations, when there are differences in the adjustment of phenotypes for covariates between studies,
• differences in genotyping measurement error between studies (when independent of phenotype, such errors tend to bias associations toward the null) and
• differences in phenotyping measurement techniques between studies, none of which are necessarily reasons to regard an observed population association as spurious.

Due to the high power of the present analysis, we found that common variants showing directionally concordant evidence for association across the three studies were often removed when we filtered variants by thresholding a statistic measuring evidence for quantitative heterogeneity in effect size (Cochran’s Q). Consequently, we devised an alternative (generalized) statistic to detect heterogeneity in effect size that we regard as implausible for genuine population associations. The three dimensional plot (Figure S2E) illustrates our approach.

##### Model Selection by Stepwise Multiple Regression

Many of our observed associations likely reflect the same underlying causal signal due to LD between the variants. For each blood index, we therefore sought to identify a parsimonious set of genetic variants explaining the genome-wide significant associations by stepwise multiple linear regression, using the fastLM implementation in the R package RcppEigen. We first partitioned the blood index-specific genome-wide significant variants into the unique minimal set of blocks such that no block could be further partitioned into subsets of variants separated by at least 10Mb. We then performed a block and blood index-specific bidirectional stepwise model selection procedure, combining the individual level data from all three studies. Every regression model we assessed included the covariates used in the original marginal analyses (i.e., study-specific principal component scores and dummy variables for recruitment center). Additionally, we included dummy variables to absorb between-study blood index variation, an adjustment which was intrinsic to the meta-analyses of marginal associations.

The stepwise procedure started with the ‘empty’ model, containing only covariates as predictors. At each step we adjusted the model by:

1. adding the unmodeled variant with the smallest p value for association with the residuals of the current model, providing that such a p value was below the genome-wide significance level (8.31x10^−9^)
2. iteratively pruning variants from the model when the p value comparing the current model with the sparser model was greater than the genome-wide significance level, with the variant corresponding to the largest such p value being pruned at each iteration.

When neither 1. or 2. were possible the procedure terminated. We modeled only the additive effects of the imputed allele dosages.

After identifying a terminal set of variants for each block, we merged the variants for each blood index across blocks and ran the same stepwise procedure but on the merged set of variants for each index, starting with the saturated model. This ensured selection of a set of variants for each index that were mutually *conditionally significant* at the genome-wide level, accounting for any residual LD over 10Mb. Although the stepwise procedure made no adjustment of p values to account for the model search, it also ignored additional strong evidence for associations from the apposition of distinct signals. Our genome-wide significance level is conservative, so the selected variants for each index are likely to represent causally distinct signals, except in regions where imputation is imprecise (where multiple variants may tag a single causal signal).

We report univariable and multivariable summary association statistics for the variants with conditionally significant associations in Table S4.

##### Consensus Set of Variants over Blood Indices

Because we performed a distinct model selection procedure for each blood cell index, a locus that was associated with multiple indices could be represented by different sentinel variants. To identify conditional variants reflecting the same signals, we clumped the selected set of variants from all indices using pairwise LD. First, we identified the set of variants considered conditionally significantly associated with at least one index after model selection. We then ‘clumped’ the variants by taking each conditionally significant variant in turn and looking for conditionally significant variants with in LD (*r*^2^ > 0.8 in the UK Biobank dataset). If other variants with *r*^2^ > 0.8 were found, then these variants were assigned to the same clump. If there were no such variants, then the new variant was assigned to a new clump. The process was repeated until each variant was assigned to a single clump. We report the summary information for each clump in Table S3. We defined the *sentinel variant* within each clump as the variant with the smallest univariable association p value across all indices.

#### Annotation of Associated Variants

##### Conditionally Significant Variant Annotation

We queried dbSNP v14 to retrieve rsIDs for all variants if available (Sherry et al., 2001). All conditionally significant variants were further annotated using the Ensembl Variant Effect Predictor (VEP) with Ensembl v83 and Gencode v24 for gene annotations (McLaren et al., 2016). Annotations were retrieved using the “most severe” option, which considers variant annotations across all genes and transcripts and selects the consequence with the greatest severity in terms of potential functional consequence (Table S4). Where the most severe consequence affected multiple genes (e.g., a variant that is intronic in overlapping genes), we listed all genes.

##### Associations with Traits and Complex Diseases

To identify whether our blood cell trait-associated signals were novel, we extracted previously reported sentinel variants associated with red blood cell traits, white blood cell traits or platelets from a recent review of published GWAS (Vasquez et al., 2016), supplemented by a literature review to identify more recent genetic studies of blood cell traits (Chami et al., 2016, CHARGE Consortium Hematology Working Group, 2016, Eicher et al., 2016, Polfus et al., 2016, Schick et al., 2016, Tajuddin et al., 2016, Ulirsch et al., 2016). We defined a locus as ‘previously reported’ if our sentinel variant, or any of its strong proxies (defined as *r*^2^ > 0.8 in European participants from the 1000 Genomes project Phase 3 or the UK10K project) had been previously reported (Table S3).

To identify whether our signals have also been associated with other traits or disease outcomes, we interrogated PhenoScanner (www.phenoscanner.medschl.cam.ac.uk), a variant-phenotype database capturing a wide range of large-scale genetic association studies, primarily from GWAS. The database includes the NHGRI-EBI GWAS Catalog (Welter et al., 2014), the GRASP database (Leslie et al., 2014), plus more than 100 publicly available sets of summary statistics from published studies. For each of our sentinel variants, we identified all proxies with *r*^2^ ≥ 0.8 in the European participants from 1000 Genomes Phase 3 or the UK10K project. We then retrieved all associations with p value < 5x10^−8^. Associations were flipped across proxies and traits to achieve a consistent direction of effect for each sentinel variant. For ease of interpretation, we split associations into three categories: expression QTL, metabolites and other traits or diseases (Table S5).

##### Annotation of Clinically Relevant Genes and Variants

First, we annotated all strong proxies (*r*^2^ ≥ 0.8) of our sentinel variants using VEP as described above and identified coding variants likely to have functional consequences (i.e., missense, nonsense, frameshift, splice site). Second, we took a systematic approach to identifying likely causal genes in regions identified to be associated with blood cell indices, using sets of genes known to cause relevant rare diseases from ClinVar and the set of genes that contain the alleles defining red cell, platelet and neutrophil antigens. ClinVar is a manually curated database of genetic variants that have evidence for a pathogenic role in human disease or phenotypes (Landrum et al., 2016).

##### Integration with BLUEPRINT Cell Type Specific Epigenetic Data

As part of the BLUEPRINT project, ChromHMM (Ernst and Kellis, 2012) was used to segment the genomes of primary blood cells into regulatory states obtained from histone marks - H3K4me3, H3K4me1, H3K36me3, H3K27ac and H3K9me3 - and DNaseI hypersensitive sites. The regulatory states are as follows: E1:*Transcription − low signal H3K36me3*, E2:*Transcription − high signal H3K36me3,* E3:*Heterochromatin − high signal H3K9me3,* E4:*Low signal,* E5:*Repressed Polycomb − high signal H3K27me3,* E6:*Repressed Polycomb − low signal H3K27me3,* E7:R*epressed Polycomb TSS − high signal H3K27me3 & H3K4me3 & H3K4me1,* E8:*Enhancer − high signal H3K4me1,* E9:*Active Enhancer − high signal H3K4me1 & H3K27Ac,* E10:*Active TSS − high signal H3K4me3 & H3K4me1,* E11:*Active TSS − high signal H3K4me3 & H3K27Ac.*

We focused on the cell types matched as closely as possible to the GWAS traits, specifically CD34-negative CD41-positive CD42-positive megakaryocytes (cord blood, 2 samples), erythroblasts (cord blood, 2 individuals), CD14-positive CD16-negative classical monocytes (venous blood, 2 individuals), mature neutrophils (venous blood, 4 individuals), mature eosinophils (venous blood, 2 individuals), naive B cells (venous blood, 3 individuals) and CD4-positive alpha beta T cells (venous blood, 4 individuals). We merged the segmentations across individuals by defining consensus states based on majority vote plus one. (e.g., for cell types measured in 2 individuals, both individuals must be called in a region as “T*ranscription High Signal - H3K36me3*” for a that to be the consensus call in the region).

We used LD score regression v1.0.0 (Finucane et al., 2015) to estimate the heritability due to common (MAF > 5%) genetic variants for each trait and to partition that heritability across regulatory states estimated from epigenomic data measured in matched cell types. We generated LD scores using the HapMap3 common variants measured in 1000 Genome Europeans (excluding Finns). We then partitioned the heritability into regulatory states estimated by the BLUEPRINT consortium.

LD score heritability estimates are based on summary statistics and are biased by genomic control adjustment. Consequently, we adjusted each raw heritability estimate by the factor

$$\lambda_{\text{META}}\frac{\frac{n_{\text{INTERVAL}}}{\lambda_{\text{INTERVAL}}}+\frac{n_{\text{UKBiLEVE}}}{\lambda_{\text{UKBiLEVE}}}+\frac{n_{\text{UKBiobank}}}{\lambda_{\text{UKBiobank}}}}{\frac{n_{\text{INTERVAL}}}{\lambda_{\text{INTERVAL}}^{2}}+\frac{n_{\text{UKBiLEVE}}}{\lambda_{\text{UKBiLEVE}}^{2}}+\frac{n_{\text{UKBiobank}}}{\lambda_{\text{UKBiobank}}^{2}}},$$

where each λ corresponds to a genomic control inflation factor (Table S2), to undo approximately the effect of our genomic control adjustments.

In order to measure systematically the statistical significance of the overlaps between our blood cell index-associated variants and BLUEPRINT epigenetic data, we used GARFIELD (Iotchkova et al., 2016b), a novel enrichment analysis approach that uses genome-wide association summary statistics to calculate odds ratios for association between annotation overlap and disease status at given genome-wide statistical significance thresholds. Tests for significance are implemented via generalized linear modeling framework accounting for LD, minor allele frequency (MAF), and local gene density. LD (r^2^) was calculated with PLINK v1.9 using variants from the combined UK10K and 1000 genomes Phase3 European cohorts in 1 Mb windows. Overlap of blood cell index-associated variants with BLUEPRINT annotations was based on genomic position overlap or LD tagging (r^2^ ≥ 0.8). Variants significantly associated with blood cell indices were ‘*greedily’* pruned by sequentially retaining the most significant variant and pruning around it (LD r^2^ ≥ 0.1) until no significant variants remained. This approach tries to ensure independence of variants in the enrichment tests, while ensuring that we retain the most significantly associated variants. We tested for enrichment all variants with MAF ≥ 1% reaching a p value of 1x10^−8^ and performed multiple testing correction based on the number of traits, segmentation states and cell types used.

##### Integration with BLUEPRINT Molecular QTL Data

Many of the common variants we discovered were non-coding (i.e., intronic, intergenic, in 5′ or 3′ untranslated regions or were just upstream or downstream of genes) suggesting they may act through regulatory mechanisms. To investigate this, we tested colocalization of the 29.5 million variants we included in our GWAS of blood indices with BLUEPRINT molecular QTL data (Table S6) using the software SMR (Summary data-based Mendelian Randomization) (Zhu et al., 2016). The BLUEPRINT QTL data consists of expression QTL (eQTL), splicing QTL (sQTL) and a histone mark H3K4me1 (hQTL) identifying sites of active or poised enhancers in ∼200 European samples (Chen et al., 2016). Data were available for monocytes, neutrophils and T cells, hence we restricted our annotation to loci that were associated with myeloid or lymphoid cell indices. SMR takes the variant with the most statistically significant association with each QTL (defined as p < 5x10^−8^), then tests whether the ratio of that variant’s effect size with the QTL against its effect size with each myeloid or lymphoid index is significant (p < 0.001). Having established the presence of a QTL and a blood cell index association at the same locus, the software then proceeds to test whether this apparently colocalized signal is the result of linkage (i.e., two independent signals in the same genomic region) or causality/pleiotropy (i.e., the same causal variant affects both the QTL and the blood cell index). This is performed via a Heterogeneity In Dependent Instruments (HEIDI) test statistic, which assesses the homogeneity of the ratio across variants in the region, with p > 0.05 indicating colocalization (Figure 6).

#### Mendelian Randomization Analysis

To evaluate the potential causal role of blood cell indices on common complex diseases, we used the set of variants we identified to perform multivariable Mendelian Randomization (MR) analysis (Table S7). MR analysis uses the random allocation of alleles at conception to obtain an “unconfounded” estimate of the association between a risk factor and an outcome, thereby avoiding the potential residual confounding and reverse causation in observational association studies. This is done by effectively treating the genetic information as a proxy for the exposure (in this case, a blood cell index). Under certain assumptions, particularly that the genetic variant only has one causal pathway to the disease which is via the blood cell index, one can assess the likely causal relationship between blood cell index and disease. Multivariable MR analysis has the added benefit that we can estimate the causal effect of each blood cell index on the outcome, conditioning on all other blood cell indices, thereby allowing us to account for the correlation between them.

Due to the high degree of genetic correlation between the blood cell indices, in particular due to the presence of calculated and compound indices, we initially selected the minimal set of indices needed to represent all 36 indices by filtering out those that were strongly correlated (r^2^ > 0.8). This left 13 sentinel indices (PLT#, MPV, PDW, HCT, MCH, RDW, RET#, IRF, MONO#, NEUT#, EO#, BASO# and LYMPH#; Table S1). We obtained variant association summary statistics (i.e., betas and standard errors) from publicly available data using the PhenoScanner (Staley et al., 2016) and ImmunoBase (www.immunobase.org). To be included, a dataset had to be large (> 5000 disease cases), have good genome coverage (> 100,000 variants), and allow identification of the direction of effect at each variant. We were able to analyze three cardiometabolic diseases (coronary heart disease, Type 2 diabetes, chronic kidney disease), five neuropsychiatric diseases (Alzheimer’s disease, bipolar disorder, cross disorder, major depressive disorder and schizophrenia) and six autoimmune diseases (asthma, celiac disease inflammatory bowel disease, multiple sclerosis, rheumatoid arthritis and Type 1 diabetes). We identified overlapping variants between our disease dataset and the list of proxies (variants with R^2^ > 0.8 with the sentinel variant) for our sentinel variants which went into the MR analysis. We then performed multivariable MR using the inverse variance weighted approach, which uses summary statistics to regress the effect of each variant on the disease outcome against its effects on the blood cell indices (Burgess and Thompson, 2015). To account for the 182 tests (13 blood cell trait indices x 14 disease outcomes), we applied a Bonferroni correction and considered associations with p < 2.7x10^−4^ (i.e., 0.05/182) to be significant.

To assess how robust our results were, we then performed sensitivity analysis using multivariable MR-Egger to test for pleiotropy. This fits the same model as the multivariable MR but allows the intercept to be freely estimated, which represents the level of unbalanced pleiotropy in the system (Bowden et al., 2015). Furthermore, for each blood cell index the regression coefficient is realigned (i.e., flipping the signs so all the associations with the index are increasing and adjusting the signs on the association with the disease accordingly to account for this) separately which ensures the intercept represents the level of unbalanced pleiotropy for that index. Since many of our most significant results involved white blood cell indices and autoimmune diseases, which both have large components of heritability driven by the MHC region, we also performed a sensitivity analysis removing the region surrounding MHC (chr6:20,000,000-40,000,000). To ensure our strong association between eosinophil count and asthma risk was genuine and not driven by a few variants with very strong effects, we removed all known variants associated with asthma at GWAS levels (p < 5x10^−8^) before repeating our analysis for asthma as a sensitivity analysis. Finally, we assessed whether our results were driven by loci that are associated with many cell lineages by repeating our analyses excluding the 42 sentinel variants representing clumps univariately associated with all five index classes (i.e., platelets, mature red cells, immature red cells, myeloid cells, lymphoid cells).

## Author Contributions

Conceptualization, J.D., D.J.R., W.H.O., A.S.B., and N. Soranzo; Methodology, W.J.A., H.E., T.J., D. Ruklisa, S.M.S., J.R.S., S.K., A.S.B., and N. Soranzo; Software, W.J.A., H.E., T.J., D. Ruklisa, M.K., J.R.S., and V.I.; Validation, W.J.A., H.E., T.J., and F.R.-M.; Formal Analysis, W.J.A., H.E., T.J., D.A., D. Ruklisa, A.L.M., M.A.K., K.K., L.B., L.G., R.P., K.W., E.W., V.I., A.S.B., and N. Soranzo; Investigation, W.J.A., H.E., T.J., D. Ruklisa, H.B., M.A.K., J.J.L., S.S., K.D., K.F., L.G., J.G., R.P., J.R.S., M.F., A.S.B., and N. Soranzo; Resources, K.B., J.R.B., O.D., S.F.G., M.H., E.M.J-M., A.K., B.K., A.M., J.M., J.H.A.M., J.O'C., N. Sharifi, S.M.S., S.T., M.v.d.E., S-Y.W., S.P.W., C.M., J.S., H.G.S., E.C.-d.-S.-P., D.J., D. Rico, A.V., L.C., B.G., L.V., T.K., D.G.-M., S.W., Y.Y., R.G., S.B., D.S.P., T.P., D.B., G.B., M.F., J.D., D.J.R., W.H.O., A.S.B., and N. Soranzo; Data Curation, W.J.A., H.E., T.J., D.A., D.Ruklisa, L.C.D., S.F.G, S.M., K.M., S.K., A.S.B. and N. Soranzo; Writing — Original Draft, W.J.A., H.E., T.J., D.J.R., A.S.B., and N. Soranzo; Writing — Review & Editing, W.J.A., H.E., T.J., T.W.K., D.J.R., W.H.O., A.S.B., and N. Soranzo.; Visualization, W.J.A., H.E., T.J., A.L.M., D.M., K.K., L.B.,V.I., and N. Soranzo; Supervision, J.D., D.J.R.,W.H.O., A.S.B., and N. Soranzo; Project Administration, D.M., A.S.B., and N. Soranzo; Funding Acquisition, J.R.B., C.M., E.D.A., M.F., J.D., W.H.O., A.S.B., and N. Soranzo.

## Supplemental Information

Table S1. Summary of Measurement Methods for Full Blood Count Indices, Related to the STAR Methods Each row of the table corresponds to one of the 36 hematological indices analyzed in the GWAS. For each model of hematology analyzer (Coulter LH700 series, used in UK Biobank, and Sysmex XN-1000, used in INTERVAL), the “Determination” column describes the technique used by the instrument to determine the value of each index. When the index is directly measured the analytic method is given, whereas when the index is derived by calculation from directly measured index, the formula used for the calculation is given. The “Previous GWAS” column denotes which hematological indices have been studied previously in GWAS.

Table S2. Characteristics of Study Samples and Genomic Inflation Factors, Related to the STAR Methods Each row of the table corresponds to one of the 36 hematological indices analyzed in the GWAS. For each blood cell index, the mean and SD after adjustment for technical covariates (but prior to adjustment for biological factors and rank inverse normalization) are given for each study (stratified by gender), as are the number of males and females included in each study after exclusions. Genomic inflation factors are given for each study and blood cell index. We also include the inflation factors estimated for both stages of genomic control correction. All inflation factors were estimated using METAL.

Table S3. Summary of Associated Loci, Related to Figure 2 and the STAR Methods Information about each of the 2,706 loci and their corresponding sentinel variants is given, ordered by chromosome and position (all coordinates are with respect to GRCh37) Locus ID is a unique identifier for each locus comprising the chromosome and an index based on position. The number of conditionally significant variants in each locus is also provided as well as the blood cell indices with which they are conditionally significantly associated and the corresponding blood cell classes. The column “Blood Index Previously Reported to be Associated with Locus” lists the blood cell indices for which a variant in the locus has been previously associated in a GWAS. The unique variant ID is constructed from the chromosome, position and the reference and alternative alleles according to the human genome reference (build 37 coordinates). Where available, the rsID is also given. Loci containing a coding variant in a gene responsible for a rare Mendelian blood-related disorder are annotated with the gene name.

Table S4. Summary of Associated Variants and Their Consequences, Related to Figures 3, 4, and 5 and the STAR Methods Summary statistics and annotations for each conditionally significant variant. Each row corresponds to a variant-trait association. Effect size estimates, standard errors, p values and -log10 (p values) from the univariable meta-analysis are given for each of the 6,736 variant-index associations, ordered by chromosome and position (all coordinates are with respect to GRCh37) as well as summary statistics from the conditional analyses. All effect size estimates (given as per-standard deviation changes) and allele frequencies correspond to additive models. The REF allele is the baseline allele and ALT allele the effect allele. For variants with ancestral allele annotations, the ancestral allele and derived allele frequency are given. Further variant annotation is provided using the Ensembl Variant Effect Predictor using the “most severe” option (McLaren et al., 2016). Genes, in which we identify variants, known to cause relevant rare diseases from the ClinVar database are also provided.

Table S5. Overlap of Loci with Previously Reported Phenotype Associations, Related to the STAR Methods For each of the 2,706 sentinel variants, previously reported associations with phenotypes and disease risks, gene expression and metabolites are listed if the variant reported was in strong LD (r2>0.8) with our sentinel variant and had a p value<5x10-8. Previous associations are reported in the following format: phenotype -log10 (p value); proxy (pubmed_id), with tissue/cell type also listed for gene expression associations. Previously reported associations were identified using Phenoscanner (http://www.phenoscanner.medschl.cam.ac.uk/), a database of variant-phenotype associations that includes the NHGRI-EBI GWAS catalogue, GRASP, and publicly available summary statistics from GWAS.

Table S6. Cellular Trait and Molecular Trait Colocalization, Related to Figure 6 and the STAR Methods Summary statistics from the colocalization analysis using SMR between neutrophil, monocyte and lymphocyte count and molecular QTL in the relevant cell types. For eQTL and sQTL in each of the three cell-types, the columns correspond to Ensembl Gene ID, the corresponding gene name (Gene Name) and information extracted from BioMart regarding the gene type (Gene Type), the Probe ID used in the BLUEPRINT QTL data (Probe ID) and the base-pair location of the probe (Probe bp), the Variant ID found in both the GWAS and BLUEPRINT QTL data (Variant ID), the variant chromosome location (Variant Chr) and base-pair location (Variant bp), the effect allele and baseline allele used in the SMR study (Effect Allele, Baseline Allele), the effect allele frequency (Effect Allele Freq.), the effect size in the GWAS study (Effect GWAS), together with the standard error and p value (SE GWAS, p value GWAS), the effect size in the BLUEPRINT QTL study (Effect QTL), together with the standard error and p value (SE QTL, p value QTL), the effect size in the SMR analysis (Effect SMR), together with the standard error and p value (SE SMR, p value SMR) as well as the p value for the heterogeneity test (p value HEIDI) and the number of variants tested in the heterogeneity test (Variant#). For the hQTL results in each cell-type, the Probe ID denotes the chromosome location as well as the start and end of the H3K4me1 or H3K27ac peak, followed by the probe chromosome location and the Histone Mark ID, which contains the same information as the probe ID. As for the eQTL and sQTL results, the columns correspond to the Variant ID found in both the GWAS and BLUEPRINT QTL data (Variant ID), the variant chromosome location (Variant Chr) and base-pair location (Variant bp), the effect allele and baseline allele used in the SMR study (Effect Allele, Baseline Allele), the effect allele frequency (Effect Allele Freq.), the effect size in the GWAS study (Effect GWAS), together with the standard error and p value (SE GWAS, p value GWAS), the effect size in the BLUEPRINT QTL study (Effect QTL), together with the standard error and p value (SE QTL, p value QTL), the effect size in the SMR analysis (Effect SMR), together with the standard error and p value (SE SMR, p value SMR) as well as the p value for the heterogeneity test (p value HEIDI) and the number of variants tested in the heterogeneity test (Variant#).

Table S7. Mendelian Randomization, Related to Figure 7 and the STAR Methods A summary of the results of Mendelian randomization (MR) analyses to estimate the causal effects of variation in 13 blood indices on predisposition to 14 diseases. The first two columns indicate the blood index and disease corresponding to the analysis. The next column is a list of indices merged into the analysed index. The subsequent columns are statistics summarizing multivariable MR analyse, which estimates the effect of all indices simultaneously, followed by sensitivity analyses. For the first of these, we remove the MHC region while in the second we removed variants known to be associated with asthma. In the third (specificity filtered) section we removed the variants associated with all blood cell index groups and in the fourth we use the MR Egger approach (Bowden et al., 2015). This method fits the same model as the multivariable MR analysis but allows the intercept of the model to be freely estimated which represents the extent of unbalanced pleiotropy. The final four columns summarise a univariable analyses where we test each blood cell index individually. For the multivariable model we used all the conditionally significant variants, in the univariable case we only used variants that were conditionally significant for that particular index. The number of these variants which are also found in each disease dataset is provided. In each case, the effect size represents the change per standard deviation increase of the transformed blood cell index in the log odds ratio of the disease risk. P values which meet the Bonferroni corrected threshold of 0.00027 are colored green while subthreshold results meeting a p value threshold of 0.05 are colored blue. For the MR-Egger sensitivity analysis, the p value for the intercept is coloured red if it meets the 0.05 threshold, indicating there is significant evidence for unbalanced pleiotropy.
